# Generation of human iPSCs from cells of fibroblastic and epithelial origin by means of the oriP/EBNA-1 episomal reprogramming system

**DOI:** 10.1186/s13287-015-0112-3

**Published:** 2015-06-19

**Authors:** Anna M. Drozd, Maciej P. Walczak, Sylwester Piaskowski, Ewelina Stoczynska-Fidelus, Piotr Rieske, Dawid P. Grzela

**Affiliations:** Department of Research and Development, Celther Polska Ltd., Milionowa 23, 93-193 Łódź, Poland; Department of Tumor Biology, Medical University of Łódź, Żeligowskiego 7/9, 90-752 Łódź, Poland

## Abstract

**Introduction:**

The prospect of therapeutic applications of the induced pluripotent stem cells (iPSCs) is based on their ability to generate virtually any cell type present in human body. Generation of iPSCs from somatic cells has opened up new possibilities to investigate stem cell biology, to better understand pathophysiology of human diseases, and to design new therapy approaches in the field of regenerative medicine. In this study, we focus on the ability of the episomal system, a non-viral and integration-free reprogramming method to derive iPSCs from somatic cells of various origin.

**Methods:**

Cells originating from neonatal and adult tissue, renal epithelium, and amniotic fluid were reprogrammed by using origin of replication/Epstein-Barr virus nuclear antigen-1 (oriP/EBNA-1)-based episomal vectors carrying defined factors. The iPSC colony formation was evaluated by using immunocytochemistry and alkaline phosphatase assay and by investigating gene expression profiles. The trilineage formation potential of generated pluripotent cells was assessed by embryoid body-mediated differentiation. The impact of additionally introduced factors on episome-based reprogramming was also investigated.

**Results:**

Reprogramming efficiencies were significantly higher for the epithelial cells compared with fibroblasts. The presence of additional factor miR 302/367 in episomal system enhanced reprogramming efficiencies in fibroblasts and epithelial cells, whereas the downregulation of Mbd3 expression increased iPSC colony-forming efficiency in fibroblasts solely.

**Conclusions:**

In this study, we performed a side-by-side comparison of iPSC colony-forming efficiencies in fibroblasts and epithelial cells transiently transfected with episomal plasmids and demonstrated that iPSC generation efficiency was highest when donor samples were derived from epithelial cells. We determined that reprogramming efficiency of episomal system could be further improved. Considering results obtained in the course of this study, we believe that episomal reprogramming provides a simple, reproducible, and efficient tool for generating clinically relevant pluripotent cells.

**Electronic supplementary material:**

The online version of this article (doi:10.1186/s13287-015-0112-3) contains supplementary material, which is available to authorized users.

## Introduction

Pluripotent stem cells have the ability to proliferate indefinitely and the potential to give rise to every other cell type present in the body. The development of nuclear reprogramming technology to derive induced pluripotent stem cells (iPSCs) from somatic cells offers the unprecedented opportunity to study stem cells in basic research and to design new patient-specific therapeutic approaches with the ultimate goal to bring them toward clinical applications.

The direct reprogramming is achieved by forced expression of a set of defined factors that are critical for the specification of pluripotent stem cell identity. Since Takahashi and colleagues [1, 2] describing that four transcription factors—Oct3/4, Sox2, Klf4, and c-Myc—were sufficient to reprogram murine and human fibroblasts, there have been a number of reports on other “gene cocktails” that can achieve the same goal in terms of conversion of somatic cells to pluripotency [3–6].

Originally, the reprogramming factors were introduced by retroviral transduction that caused the genomic integration of delivered transgenes. Although this method is simple and efficient, the concern of clinical application of iPSCs established in such a manner involves the risk of insertional mutagenesis and oncogenic potential of some factors, especially Klf4 and c-Myc. To comprise high efficiency and safety of integrative vectors, excisable systems have been developed. Lentiviruses with loxP site introduced into their 3′ long terminal repeat (3′ LTR) retained the ability to integrate into the host DNA, resulting in efficient and long-term transgene expression. With application of Cre recombinase, it is possible to excise floxed reprogramming genes after the generation of iPSCs [7, 8]. Another approach involves the use of transposons, which have been shown to be equally efficient to the abovementioned viruses regarding long-term transgene expression [9, 10]. However, none of the genome-integrating vectors can be regarded as completely safe, because of DNA footprint left after transposon or Cre/loxP-based viral excision or because of possible homologous recombination events between closely positioned identical sequences that could lead to DNA deletion and genomic rearrangements.

The concerns about genome integrity in the process of generation of iPSCs led to the exploration of non-integrating methods for factors delivery. Such approaches involve the use of polycistronic minicircles [11], non-integrating DNA viruses [12], plasmid transfections [13, 14], or the delivery of the reprogramming factors in the form of cell-penetrating proteins [15]. Though safer, the application of these methods heavily compromises iPSC generation in terms of reprogramming efficiency. Among other integration-free methods, Sendai virus-based vectors have been used for efficient derivation of human iPSCs [16]. The inherent features of Sendai virus include the cytoplasmic retention and the existence of viral genome in the form of RNA during the entire replication process. However, because the Sendai virus has been shown to possess strong immunogenic potential and because of the long-term presence of the virus in infected cells, the clinical application of iPSCs generated by means of Sendai vector would require labour-intensive viral particle removal.

Other recent methods in the generation of iPSCs include the expression of reprogramming factors delivered with episomal DNA vectors. Episomes are non-integrating and non-viral, plasmid-based vectors and therefore are safe to use and inexpensive. In addition, only a small number of transfections is required, and the chance of random integration into the genome of transfected host cells remains low. For long-term heterologous gene expression, episomal vectors based on components of BK virus, bovine papilloma virus-1, SV40 virus, Epstein-Barr virus (EBV), and scaffold/matrix attachment region (S/MAR) elements have been used, as they offer attractive alternatives to transient transfections and integrative methods (reviewed in [17, 18]). So far, only episomal vectors based on components of EBV have been used to obtain pluripotent cells. EBV-based episomes are DNA plasmids that carry the origin of replication (oriP) element and the *cis*-acting EBV nuclear antigen-1 (EBNA-1). These two are the only components derived from EBV. OriP is composed of the family of repeats (FR) and dyad symmetry (DS) elements. DS region is bound through the helix-turn-helix domain of EBNA-1 protein required for replication of episome in mammalian cells. In primates, EBNA-1 binding to the oriP origin tethers the episomal DNA to the cell chromosomes, allowing EBNA-1-mediated amplification and episome segregation during each cell division. Owing to the non-perfect nature of oriP/EBNA-1 partitioning, episomes are gradually lost at ratios of 2–8 % per cell division in the absence of selection pressure. Eventually, this would lead to depletion of episomes carrying reprogramming factors after 2–3 months of iPSC culture.

The applicability of the oriP/EBNA-1-based system to generate iPSCs was demonstrated for the first time for derivation of pluripotent cells from human dermal fibroblasts [19]. In addition to EBV-derived elements, vectors described in this study comprised OCT3/4, SOX2, NANOG, KLF4, LIN28, C-MYC, and large antigen T of SV40 virus, driven by moderate human EF1α promoter. Obtained iPSC generation efficiencies were two orders of magnitude lower than reported for lentiviral vectors and needed the supplementation of small chemical molecules to achieve a sufficient number of iPSC colonies.

Further improvements of episomal system involved extensive vector redesigning [20, 21]. For this purpose, the size of the episomal vectors has been reduced and their number has been increased and this probably resulted in easier vector delivery through cell membrane. Reprogramming factors were connected via 2A self-cleaving peptides instead of internal ribosome entry site (IRES) elements, SV40LT and NANOG were excluded, C-MYC was replaced with more potent and specific L-MYC, and either short hairpin RNA (shRNA) or dominant negative mutant was used for p53 suppression. The expression level of EBNA-1 antigen appeared to be a limiting factor of the episome-based reprogramming since the introduction of additional vector carrying EBNA-1 under control of strong promoter enhanced the iPSC-forming efficiency. Additionally, the use of more potent CAGGS promoter instead of moderate EF1α promoter, together with aforementioned alterations, led to a more than 10-fold increase of reprogramming efficiency.

Besides the reprogramming vectors used for generation of iPSCs and composition of “genes cocktail” they carry, the additional issue that needs to be considered for efficient reprogramming process is the source of donor cells. A number of iPSC lines have been derived from different human cells, indicating that reprogramming factors have the ability to establish pluripotency state from multiple donor cells [22–25]. However, depending on cells isolated from different tissues, the actual reprogramming is achieved with different efficiencies and varying temporal kinetics.

Optimally, for generation of iPSCs, the sample collection from patients for subsequent reprogramming should be performed in a negligible invasive manner. Pluripotent cells were derived from peripheral blood mononuclear cells with the application of Sendai virus [26]. Blood is an easily accessible source of cells for reprogramming, but the collection procedure is still considered invasive to some degree. Another source of easily accessible material are follicles from hairplucks. It has been shown [27] that keratinocytes established from isolated hair follicles, after transduction with retroviral vectors carrying OSKM (Oct3/4, Sox2, Klf4, and Myc) factors, reprogram 100 times more efficiently than fibroblasts. Successful iPSC generation was also achieved by using exfoliated renal epithelial cells isolated from urine. Also, the isolation procedure is completely non-invasive and provides no significant inconvenience to the donor, and the urine-derived cells exhibit remarkably high iPSC colony formation efficiency, up to 4 % as determined by using retroviral reprogramming [28].

In this study, we made an attempt to reprogram cells of various origin by using oriP/EBNA-1 episomal system and to compare efficiencies of the process. To reprogram cells effectively, we aimed to optimize transgene delivery conditions and to include factors enhancing iPSC colony formation. We believe that findings described in our study may become useful in other experiments that involve generating iPSCs.

## Methods

### Ethics statement

Written approval for human cell sample collection and subsequent generation of iPSC lines in this study in adherence to the local ethical regulations was obtained from the Bioethical Committee of the Regional Medical Chamber in Łódź (approval number K.B. 17/13 from 13 November 2013), and written informed consent was provided by each individual patient.

### Reagents

Unless stated otherwise, all chemicals were from Sigma-Aldrich (St. Louis, MO, USA). Cell culture media were purchased from Life Technologies (Carlsbad, CA, USA). Restriction endonucleases, polymerases, and DNA-modifying enzymes were from New England Biolabs (Ipswitch, MA, USA).

### Episomal reprogramming plasmids

Episomal vectors used in this study were purchased from Life Technologies as Epi5™ Episomal iPSC Reprogramming Kit consisting of a mixture of oriP/EBNA1-based expression constructs described earlier by Okita et al. [21]. Plasmid information and sequences are available from Addgene (Cambridge, MA, USA) [29] under accession numbers #41813, #41814, #41855, #41856, and #41857.

### Vector construction

pCXB-DEST construct, a DNA fragment containing constitutive CAGGS promoter, mammalian polyadenylation signal, *Escherichia coli* pUC replication origin, and β-lactamase gene was polymerase chain reaction (PCR)-amplified from pCXB-EBNA1 plasmid with Q5 DNA polymerase with pCXB Fwd and pCXB Rev primers. The resulting 4.4-kbp PCR product was isolated from the agarose gel with a QIAquick DNA Gel Extraction Kit (Qiagen, Venlo, Limburg, The Netherlands) and ligated with Gateway Rf.A cassette encompassing attR1 and attR2 recombination sequences, chloramphenicol resistance gene, and ccdB negative selection marker. The correct orientation of the ligated insert was determined by restriction digest and DNA electrophoresis.

The oriP/EBNA-1-based episomal expression vector *pCE-DEST* was generated by shuttling SnaBI/HindIII-digested fragment from pCXB-DEST plasmid into pCE-hUL backbone digested with the same enzymes.

To create *pCE-mSEAP* vector driving the expression of murine secreted embryonic alkaline phosphatase, the mSEAP coding sequence was PCR-amplified from pCpGfree-mSEAP plasmid (InvivoGen, San Diego, CA, USA) with mSEAP attB1 and mSEAP attB2 primers. Obtained PCR product was introduced into pENTR/zeo-mSEAP vector by means of Gateway BP Clonase II enzyme and pDONR/zeo plasmid (Life Technologies). The transfer of the mSEAP fragment from the ENTRY construct into the final pCE-mSEAP vector was done by using Gateway LR Clonase II enzyme (Life Technologies).

To generate *pCE-mCherry-miR302/367* vector, encoding the fluorescent marker and miR 302/367 cassette under CAGGS promoter, mCherry coding sequence was synthesised by GeneArt AG and PCR-amplified with FP attB1 and FP attB2 primers by using Q5 DNA polymerase. PCR product was shuttled into pDONR/zeo plasmid, resulting in pENTR/zeo-mCherry construct. After that, the miR 302/367 fragment was amplified from the genomic DNA of SNL cells (Cell BioLabs, San Diego, CA, USA) with miR 302/367 Fwd and miR 302/367 Rev primers and combined to DNA fragment (amplified from pENTR/zeo-mCherry by pENTR/zeo-mCherry Fwd and pENTR/zeo-mCherry Rev primers) by means of Gibson assembly reaction (New England Biolabs). The resultant sequence from ENTRY vector was shuttled into the expression construct with use of Gateway LR Clonase II enzyme (Life Technologies). To create *pCE-shMbd3* construct for downregulation of Mbd3 protein, Mbd3sh top and Mbd3sh bottom primers were assembled into double-stranded DNA (dsDNA) oligonucleotide, which was then ligated with pENTR/U6 vector, between U6 promoter and PolIII terminator, in accordance with the protocol of the manufacturer (Life Technologies). The resultant construct was transferred into episomal vector pCE-DEST by means of Gateway LR Clonase II enzyme (Life Technologies). All vectors were verified by DNA sequencing and restriction digest analysis. DNA sequences of oligonucleotides used for cloning purposes are included in Additional file 1: Table S1.

### Cell culture

#### Human embryonic stem cells

BG01V line was purchased from AMS Biotechnology (Abingdon, UK). Cells were maintained in Essential 8 medium supplemented with penicillin G (100 U/ml), streptomycin (100 μg/ml), and amphotericin B (0.25 μg/ml) on cell culture plates coated with Geltrex basement membrane matrix (1 μg of protein per 1 cm^2^).

#### Neonatal fibroblasts

BJ and HFF-1 foreskin fibroblasts were obtained from ATCC (Manassas, VA, USA). Newborn human foreskin fibroblasts were purchased from AMS Biotechnology. Cells were cultured in Dulbecco’s modified Eagle’s medium (DMEM) supplemented with 10 % foetal calf serum (FCS), penicillin (100 U/ml), streptomycin (100 μg/ml), and amphotericin B (0.25 μg/ml).

#### Adult scar tissue fibroblasts

Pieces (1 cm^2^) of the scar tissue were rinsed with phosphate-buffered saline (PBS) (GE Healthcare, Little Chalfont, Buckinghamshire, UK) and digested with 0.5 % Collagenase IV (Sigma-Aldrich) in DMEM for 4 h with agitation at 37 °C. The digested skin tissue was filtered through 70- and 100-μm cell strainers (BD Biosciences, Franklin Lakes, NJ, USA) and centrifuged for 10 min at 250×*g*. The supernatant was discarded and the cell pellet was suspended in fresh DMEM and counted on a ADAM-MC Cell Counting System (NanoEnTek, Seoul, Korea). Finally, cells were plated on cell culture dishes at desired density and maintained in DMEM HG (with 4.5 g/l of glucose) supplemented with 10 % FCS, 1 mM of Glutamax (Life Technologies), 1 × non-essential amino acids (NEAAs) (Life Technologies), and antibiotics.

#### Urinary epithelial cells

Epithelial cells were isolated from urine samples in accordance with the protocol described previously [28]. Briefly, approximately 200 ml of urine was collected into a sterile container. It was then transferred to 50-ml tubes and centrifuged at 400×*g* for 10 min at room temperature. The supernatant was removed and cell pellet was suspended in 50 ml of the washing buffer (PBS supplemented with penicillin/streptomycin/amphotericin B). The supernatant was discarded, and cells were suspended in primary medium (DMEM/F12 supplemented with 10 % FCS, 100 U/ml of penicillin, 100 μg/ml of streptomycin, 10 ng/ml of epidermal growth factor, 5 μg/ml of insulin, 0.5 μg/ml of epinephrine, 36 ng/ml of hydrocortisone, 5 μg/ml of transferrin, and 4 pg/ml of triiodo-L-thyronine) and plated on gelatine-coated cell culture plates. Cells were maintained in primary medium for 3 days; afterwards, non-adherent cells were removed, and the culture medium was replaced with REBM medium supplemented with a Renal Cell Growth Medium (REGM) SingleQuot Kit (Lonza, Basel, Switzerland).

#### Amniotic fluid cells

Amniotic fluid samples were obtained from women undergoing amniocentesis for prenatal diagnosis at 16–19 weeks of pregnancy. Amniotic fluid (2–3 ml) was centrifuged for 10 min at 250×*g*. Cell pellets were resuspended in BIOAMF™-2 Medium (Biological Industries, Kibbutz Beit-Haemek, Israel) and incubated at 37 °C. After 7 days, non-adherent cells were removed, and the remaining adherent cells were further cultured in the medium.

### DNA transfections

For experiments involving DNA transfections of human cells, plasmids were propagated in OmniMAX 2 or Mach1 (Life Technologies) *E. coli* strains and prepared by using a NucleoBond PC500 plasmid maxiprep kit (Macherey-Nagel, Düren, Germany). DNA quality and concentration were determined on NanoPhotometer (Implen, München, Germany), and plasmids were stored at −20 °C.

Linear polyethylenimine (PEI) (molecular weight of 25,000) was purchased from Polysciences Inc. (Warrington, PA, USA) and dissolved in sterile water. For DNA transfections using PEI reagent, PEI stock solution was diluted in 100 μl of 150 mM NaCl. The plasmid DNA (1 μg) was diluted in 100 μl of 150 mM NaCl in a separate tube. The contents of the tubes were combined by gentle pipetting and incubated at room temperature for 30 min. The resulting 200 μl of solution was used for the transfection of cells grown on a six-well plate in 2 ml of medium.

For plasmid transfections with FuGENE6 and FuGENE HD (Promega, Madison, WI, USA), transfection reagent was diluted in pre-warmed Opti-MEM medium and incubated for 5 min at room temperature. Plasmid DNA was added to the mixture up to a total volume of 100 μl and incubated for 30 min. The solution of the complexes was added in a dropwise manner directly to cells grown in one well of a six-well plate in 2 ml of medium.

For transfections with Lipofectamine LTX reagent (Life Technologies), plasmid DNA was diluted in 125 μl of Opti-MEM reduced serum medium and combined with solution of Lipofectamine LTX/PLUS reagents diluted in 125 μl of Opti-MEM. The Lipid/DNA/PLUS Reagent complexes were allowed to form by incubation of the reaction mixture at room temperature for 30 min. Afterwards, the mixture of DNA/Lipid complexes was added in a dropwise manner to cells grown in one well of a six-well plate in 2 ml of medium.

### Secreted alkaline phosphatase reporter assay

Cells were transfected with indicated amounts of pCE-secreted alkaline phosphatase (pCE-SEAP) plasmid and FuGENE6 reagent. The following day, medium was changed, and after two more days, culture medium was collected and separated from cellular debris by centrifugation. Supernatants were heated to 65 °C in order to inhibit endogenous alkaline phosphatase. To determine enzyme activity, a SEAP Reporter Assay Kit (InvivoGen) was used. Briefly, 10 μl of cell culture medium was mixed with 25 μl of 1× dilution buffer, 50 μl of 1× assay buffer, 10 μl of 100 mM L-homoarginine, and 20 μl of staining solution (p-nitrophenyl phosphate, 75 μM). Samples were incubated at 37 °C for 1 h before the absorbance was measured at 450 nm.

### xCELLigence impedance analysis of the transfection-related cytotoxicity

The Real-Time Cell Analyzer (RTCA) xCELLigence (Roche, Basel, Switzerland) was used to determine cell viability after the transfection. Cell culture medium (50 μl) was added into each well of E-plate 16. The plate was allowed to equilibrate for 5 min at 37 °C, connected to RTCA system, and checked for proper electrical connections. The background impedance was measured for 60 s. Cells used for the experiment were grown and expanded in tissue culture vessels. After reaching 50 % confluence, cells were rinsed with PBS and detached from plates by treating them briefly with Accutase. Single cell suspension was prepared and cells were counted on an ADAM-MC Cell Counting System (NanoEnTek). Volumes were adjusted to obtain 1.2×10^5^ cells/ml suspension. Fifty microliters of each cell suspension was added to medium-containing wells on E-plate 16, resulting in a density of 6000 cells per well. On the next day, the culture medium was changed, and cells were transfected with 100 ng of plasmid DNA. Finally, the cell adhesion and viability were monitored every 60 min for a period of up to 72 h post-transfection with the sensor electrode arrays of the E-plate 16. The electrical impedance was quantified by the RTCA system as cell index (CI) values. CI is a unitless parameter derived from relative changes in measured impedance that is obtained in the presence and absence of cells. As the electrical impedance depends on the number of cells attached to the electrode as well as the dimensional changes of the attached cells, CI values delineate the physiological state of analysed cells.

### Generation of induced pluripotent stem cells

Unless stated otherwise, to generate iPSC lines from cells of different origin, human cells were seeded at a density of 8×10^4^ per well of a six-well plate coated with Geltrex basement matrix, with 2 μg of protein for each 1 cm^2^ area of the cell culture vessel. Cells were maintained in primary growth medium suitable for each cell type (DMEM 10 % FCS for BJ cells and scar tissue fibroblasts, BIOAMF for amniocytes, and REGM for urinary epithelial cells). After overnight incubation, culture medium was replaced with a fresh one, and cells were transfected with 2 μg of episomal plasmids (400 ng of each: pCE-hOct3/4, pCE-hSK pCE-hUL, pCE-mp53DD, and pCXB-EBNA1) by using FuGENE6 transfection reagent at a 3:1 reagent-to-DNA ratio. The next day, culture medium was replaced with TeSR-E7 medium (StemCell Technologies, Vancouver, BC, Canada), and the transfection was conducted as previously. During the reprogramming process, transfected cells were cultured in TeSR-E7, and the medium was changed every other day up to 2 weeks post-transfection. On day 15 after initial transfection, culture medium was changed to Essential 8. The medium was replaced daily for the next 3 weeks. Within 20 to 30 days post-transfection, iPSCs expanded to a size appropriate for transfer. Colonies were transferred onto new culture dishes covered with Geltrex matrix by using a pipette tip. An hour before the procedure, 10 μM Y-27632 was added to the culture medium. The aforementioned were further propagated and maintained in Essential 8 medium.

### Human embryonic stem cell and induced pluripotent stem cell culture

Human embryonic stem cells (hESCs) and iPSCs were routinely cultured under low oxygen conditions (5 % O_2_/5 % CO_2_) in chemically defined Essential 8 medium supplemented with penicillin G (100 U/ml), streptomycin (100 μg/ml), and amphotericin B (0.25 μg/ml) on cell culture plates coated with Geltrex basement membrane matrix (1 μg of protein per 1 cm^2^). Cells were passaged twice a week when they reached 60–70 % confluence. Cells were incubated in Essential 8 medium supplemented with 10 μM Y-27632 or 10 μM Blebbistatin for 1 h before passaging and colony expansion. After rinsing with PBS, cells were incubated with 0.5 mM ethylenediaminetetraacetic acid (EDTA) in ion-free PBS for 5–8 min at room temperature. EDTA solution was removed when cells started to dissociate, and the process was terminated by adding Essential 8 medium. Clumps of cells were transferred into fresh culture vessels coated with Geltrex matrix. After the passage, iPSCs and embryonic stem cells (ESCs) were maintained in Essential 8 medium supplemented with 10 μM Y-27632 or Blebbistatin for 24 h. Afterwards, the aforementioned chemicals were withdrawn and the medium was changed daily.

### Immunofluorescence analysis

For immunofluorescence studies, cells were cultured on gelatin-, poly-L-lysine-, or Geltrex-coated glass coverslips. For all stainings, cells were fixed with 4 % paraformaldehyde (PFA) in PBS for 20 min at room temperature. After fixation, samples were washed once with TBS-T (Tris-buffered 0.9 % saline, pH = 7.2 supplemented with 0.05 % of Tween-20) and permeabilized for 10 min in TBS-T/0.25 % Triton X-100. Following permeabilization and three washing steps with TBS-T, samples were incubated for at least 15 min in blocking solution (5 % bovine serum albumin or 10 % donkey serum in TBS-T). Cells were incubated with primary antibody for 1 h at room temperature. After three washing steps with TBS-T, samples were incubated with Alexa-conjugated secondary antibody (Life Technologies) and counterstained with 4’,6-diamidino-2-phenylindole (DAPI) (50 ng/ml) for 1 h at room temperature. Glass coverslips were washed in deionized water to remove salt excess and mounted in 10 % polyvinylalcohol in 25 mM Tris–HCl (pH 8.7) with 5 % glycerol and 2.5 % 1,4-diazobicyclo[2,2,2]-octane. Samples were examined on an Eclipse Ci-S epifluorescence microscope (Nikon, Tokyo, Japan) equipped with a DS-5Mc colour CCD (charge-coupled device) camera (Nikon). Images in blue channel (350/50ex, 400lp, 460/50em), green channel (480/40ex, 510lp, 535/50em), and red channel (560/40ex, 585lp, 630/75em) were acquired with NIS-Elements 4.0 and processed with ImageJ software. Antibodies used for immunofluorescence analysis are listed in Additional file 2: Table S2.

### Alkaline phosphatase staining

Cultures of presumptive iPSCs and hESCs were fixed with 4 % PFA in PBS for 30 min. Cells then were washed with TBS (2×5 min) and stained with 5-bromo-4-chloro-3-indolyl phosphate/nitro-blue tetrazolium (BCIP/NBT) substrate solution (0.02 % BCIP, 0.03 % NBT, 5 mM MgCl_2_ in 150 mM TBS, pH 9.5) at 37 °C until the purple colour emerged, washed with distilled water, and dried.

### Reprogramming efficiency calculation

To compare iPSC generation efficiencies from various cells and for the purpose of this study, the reprogramming efficiency was defined as the ratio of the number of colonies positive for alkaline phosphatase activity to the total number of cells used in the reprogramming experiment.

### Embryoid body formation

iPSC and hESC colonies cultured for 24 h in Essential 8 medium supplemented with 10 μM of Y-27632 were dissociated to a single cell suspension by StemPro Accutase (Life Technologies) and counted. Embryoid bodies (EBs) composed of approximately 5000 cells were formed by using AggreWell 800 plate (Stem Cell Technologies) by seeding 1.5×10^6^ cells per well. After 2 days, EBs were transferred to low-adhesion non-coated polystyrene plates (Nunc, part of Thermo Scientific, Waltham, MA, USA) and maintained in suspension culture in Essential 6 medium for 7–10 days. For endoderm differentiation, the EBs were transferred into RPMI medium supplemented with 2 % FCS, 100 ng/ml of Activin A, and 3 μM of CHIR99021 for 48 h. Mesoderm differentiation was initiated with DMEM/F12 medium supplemented with 10 % FCS, 500 μM L-ascorbic acid, and 100 μM β-mercaptoethanol. For ectoderm induction, the EBs were cultured in DMEM/F12 medium with N2/B27 supplements, 100 μM β-mercaptoethanol, and 1 × NEAAs. After the precondition step, the EBs were transferred onto poly-L-lysine-coated glass coverslips and cultured for 7 days in Essential 6 medium, fixed, and analysed for expression of the three embryonic germline layers markers by immunofluorescence.

### Gene expression measurements

Pluripotency and differentiation potential was assessed by using the TaqMan human pluripotent stem cell (hPSC) Scorecard kit (Life Technologies), a predesigned gene expression quantitative PCR (qPCR) assay consisting of the TaqMan probes specific for reference markers. The contents of the Scorecard panel are based on the work of Bock et al. [30] and were validated against multiple hESC and iPSC lines. Reference standards include 94 validated controls, housekeeping, self-renewal, and lineage-specific genes. The total RNAs from cells were isolated with a NucleoSpin TriPrep kit (Macherey-Nagel) as indicated in the protocol of the manufacturer. RNA concentration was measured spectrophotometrically, and 1 μg of total RNA was reverse-transcribed into single-stranded cDNA by using random hexamer primers from Thermo Scientific and M-MuLV reverse transcriptase. Quantitative real-time PCR was carried out by using TaqMan Fast Advanced Master Mix (Life Technologies) on the StepOne Plus real-time PCR System (Life Technologies) in accordance with the instructions of the manufacturer. The resulting expression data set was analysed by using cloud-based TaqMan hPSC Scorecard software (Life Technologies) in order to compare acquired expression pattern with assay-included reference standards.

### Statistical analyses

The reporter gene studies were carried out at least three times and presented as the average values ± standard error of the mean (SEM). The SEM was calculated by dividing the standard deviation by the square root of the counts. Difference between samples was compared by the two-tailed Student test and was considered significantly different at a *P* value of less than 0.05.

## Results

### Optimization of transfection conditions for efficient episomal delivery

As the efficient gene transfer is a prerequisite for successful reprogramming of somatic cells into iPSCs, we decided to optimize conditions for transfer of episomes carrying reprogramming factors.

First, we compared transfection efficiencies in neonatal fibroblasts by using commercially available transfection reagents in order to identify compounds providing the highest transfection efficiencies without compromising cell viability. Human fibroblasts (BJ cells) were seeded in a 12-well plate on glass coverslips at a density of 1×10^5^ cells per well. Next, they were transfected with 1 μg of plasmid DNA pCE-EmGFP (carrying fluorescent reporter) by using PEI, Lipofectamine, FuGENE6, and FuGENE HD at 0.5, 1, 3, and 6 reagent volume-to-DNA ratios. Twenty-four hours later, fibroblasts were analysed for transgene expression. The highest expression levels were achieved with PEI and FuGENE6 reagents, as presented in Additional file 3: Figure S1.

Although the PEI gave the highest transfection efficiencies, we observed the significant cell death caused by this compound, whereas there was no such effect for other transfection reagents. Therefore, we decided to use FuGENE6 for further experiments.

In subsequent optimization studies, we used BJ line as well as cells derived from scar tissue samples, amniotic fluid, and urine samples. To quantify reporter gene expression, we used pCE-mSEAP construct coding murine secreted alkaline phosphatase under the CAGGS promoter. Cells were seeded on four-well plates at a density of 2×10^4^ cells per well and transiently transfected with 0.5 μg of the DNA. Four plasmid:FuGENE ratios were tested: 0.75, 1.5, 3, and 4.5. The next day, cell culture medium was collected and examined for the alkaline phosphatase activity. Three independent repetitions were carried out, and the result of the experiment is presented in Additional file 4: Figure S2A.

Additionally, we performed monitoring studies of cell number and their adhesion by using xCELLigence system to determine the cells’ viability after transfections. Fibroblasts, amniocytes, and urinary-derived epithelial cells were seeded on the E-plates at a density of 6000 cells per well with 100 ng of pCE-mSEAP using the same ratios as for alkaline phosphatase activity studies. Monitoring of the changes of cell number was carried out for 72 h, and results of this experiment are presented in Additional file 4: Figure S2B.

The above experiments demonstrated that, from all tested transfection reagents, FuGENE6 provided the highest transfection efficiencies and caused the lowest harm to the cells. In addition, 3:1 (FuGENE6 volume to micrograms of the DNA) transfection ratio was found to be optimal in terms of both transgene expression and transfection-related cytotoxicity. Therefore, for subsequent reprogramming studies, we decided to follow these parameters.

### Derivation of induced pluripotent stem cell lines from cells of different origin

To investigate the utility of the oriP/EBNA1 episomal system as a tool for generation of iPSCs from differentiated cells of various origin, we conducted a side-by-side comparison of reprogramming efficiencies by using cells derived from skin tissue, amniotic fluid, and urinary samples. Cells belonged to the two distinct groups based on their morphology. BJ line derived from neonatal foreskin tissue and adult scar tissue cells exhibiting typical spindle-shaped bipolar or multipolar fibroblastic morphology. On the other hand, cells isolated from amniotic fluid and urine were tightly packed and polygonal in shape and had a regular appearance displaying morphological features characteristic for epithelial cells. Reprogramming factors included OCT3/4, SOX2, KLF4, L-MYC, and LIN28. In addition, we used mutated p53 protein to overcome reprogramming barriers (like cellular senescence) and EBNA-1 protein of Epstein-Barr virus to facilitate the long-term presence of introduced episomal vectors.

**Fig. 1:**
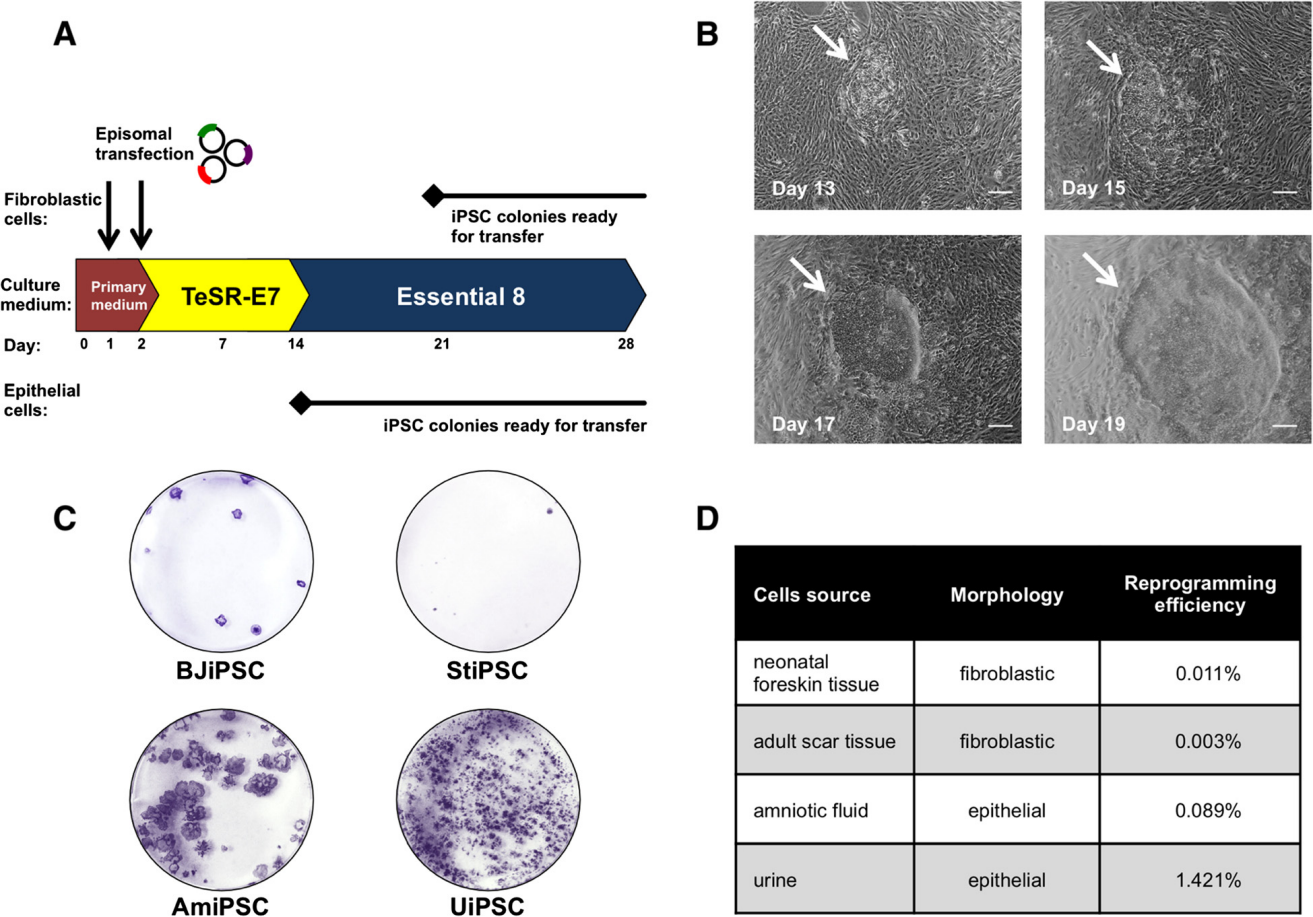
Generation of human induced pluripotent stem cells (iPSCs) with episomal vectors. (**a**) Schematic diagram of the protocol used to obtain iPSCs by transient introduction of episomes carrying OCT3/4, SOX2, KLF4, L-MYC, and LIN28 transcription factors and dominant negative mutant of p53 protein into human somatic cells. (**b**) Exemplary result of iPSC colony formation. Changes in cellular morphology during iPSC colony formation in urinary epithelial cells between days 13 and 19 after the initial transfection are shown. Arrows indicate the emerging embryonic stem cell-like colonies throughout the reprogramming experiment. Scale bar = 100 μm. (**c**) Alkaline phosphatase stainings of iPSCs generated from neonatal fibroblasts, adult scar tissue fibroblasts, amniotic fluid cells, and urinary epithelial cells at days 21, 28, 28, and 14, respectively, after the initial transfection with episomal plasmids. BJiPSCs (iPSCs derived from BJ cells), StiPSCs (iPSCs derived from scar tissue fibroblasts), AmiPSCs (amniocyte-derived iPSCs), and UiPSCs (iPSCs reprogrammed from urinary epithelial cells) are shown. (**d**) Efficiencies of iPSC colony formation in fibroblastic and epithelial cells transfected with reprogramming vectors. The efficiencies were calculated as number of colonies positive for alkaline phosphatase activity divided for total cell number plated for the experiment

Cells were seeded on Geltrex-coated plates in densities of 8×10^4^ cells per well and the next day were transfected with 2 μg of the mixture of episomal plasmids. The transfection was repeated the following day, and the cell culture media were replaced by TeSR-E7, which was changed every other day for 2 weeks. After that, TeSR-E7 was switched to Essential 8, and cells were maintained in this culture medium for the rest of the experiment. The time schedule and culture conditions of this experiment are presented in Fig. 1a. iPSC colonies started to form from the 14th day after the initial transfection in the case of epithelial cells (amniocytes and urinary cells) and after approximately 21 day for fibroblastic cells. The colonies were distinguishable by their characteristic morphology, visible as a small round cells growing in clusters with clear edges. The appearance of ESC-like cells during the experiment is shown in Fig. 1b. iPSC colonies obtained from reprogrammed urinary cells (UiPSCs) were mechanically picked up from the plate at day 14. Induced pluripotent cells derived from BJ fibroblasts (BJiPSC) were transferred after 3 weeks from the beginning of the experiment, whereas the amniocytes and scar tissue-derived iPSC colonies (AmiPSC and StiPSC) were transferred to a fresh Geltrex-coated plates 4 weeks after the first transfection with episomal reprogramming vectors. Plates with remaining colonies were fixed with paraformaldehyde and stained for the alkaline phosphatase activity (marker of pluripotent cells) as shown in Fig. 1c. Colonies positive for alkaline phosphatase were counted, and the reprogramming efficiencies for each of the studied cells were calculated as presented in Fig. 1d. The reprogramming efficiencies from independent experiments are presented in Additional file 5: Table S3.

### Characterization of the functional properties of established induced pluripotent stem cell lines

**Fig. 2:**
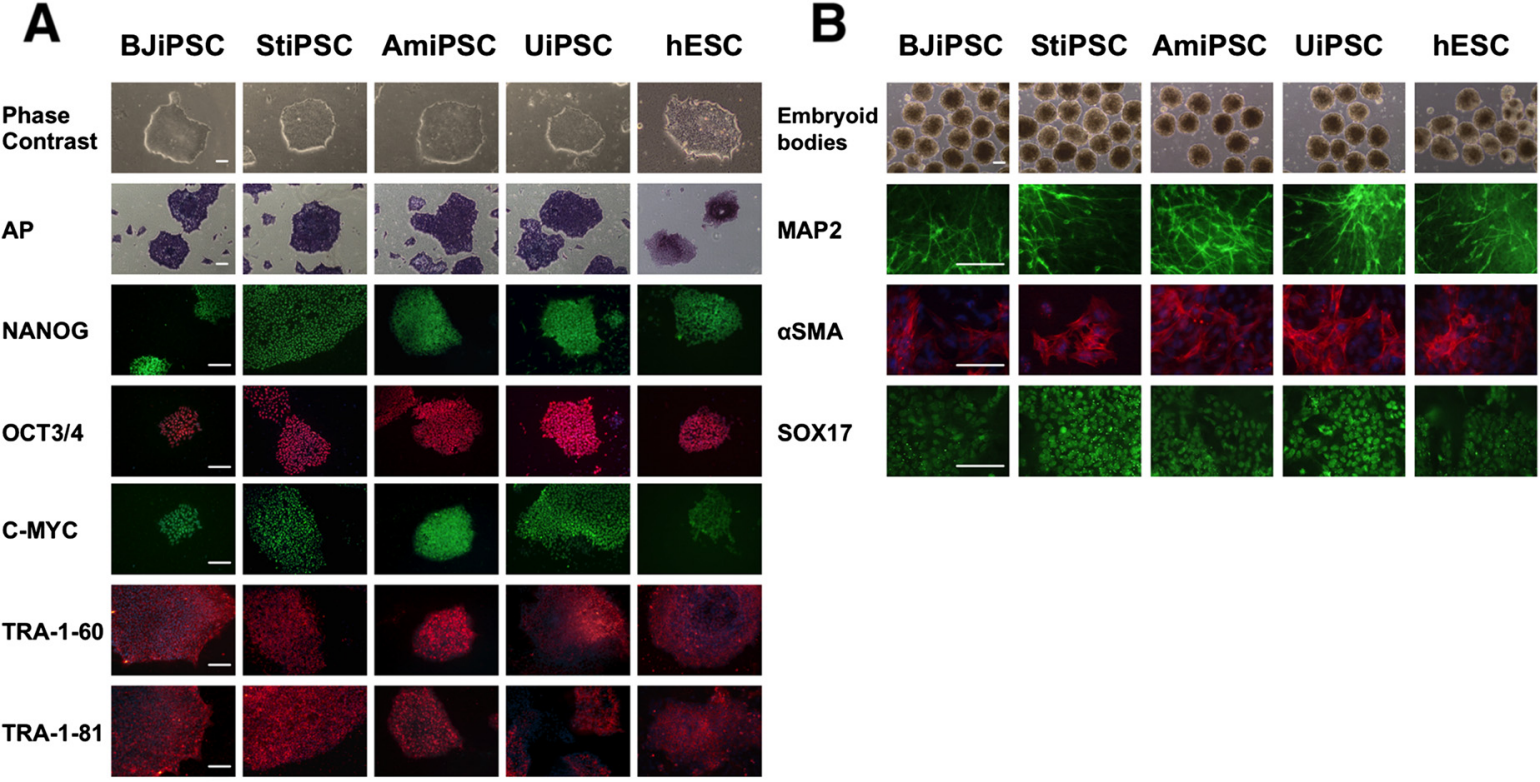
Characterization of induced pluripotent stem cell (iPSC) derived from fibroblastic and epithelial cells and their functional properties. (**a**) Detection of pluripotency-associated markers. The representative images of human iPSC colonies in established lines show a typical embryonic stem cell-like morphology and demonstration of alkaline phosphatase (AP) activity in studied cells. iPSCs derived from neonatal and adult fibroblasts as well as epithelial cells were positive for NANOG, OCT3/4, and C-MYC transcription factors and displayed TRA-1-60 and TRA-1-81 expression on their surface. Human embryonic stem cell (hESC) line BG01V was used as a positive control. Shown are representative immunofluorescence images captured by using an Eclipse Ci-S epifluorescence microscope with 20× objective. Scale bar = 100 μm. (**b**) Embryoid body (EB)-mediated multilineage differentiation of episomally generated iPSC lines. Phase-contrast images showing the EBs formed from studied iPSC lines. EBs were spontaneously differentiated and examined for the presence of markers of the three germ layers. Immunostainings show cells positive for ectodermal marker MAP2, the mesodermal marker alpha-smooth muscle actin (αSMA), and the endodermal marker SOX17. The trilineage potential is additionally evidenced by the presence of cells with morphology characteristic for neurons and cytoskeleton typical for mesodermal cells. Scale bar = 100 μm. *AmiPSC* amniocyte-derived induced pluripotent stem cell, *BJiPSC* induced pluripotent stem cell derived from BJ cell, *StiPSC* induced pluripotent stem cell derived from scar tissue fibroblast, *UiPSC* induced pluripotent stem cell reprogrammed from urinary epithelial cells

ESC-like colonies generated from neonatal and adult fibroblasts, amniocytes, and urinary cells were expanded on Geltrex-coated plates and used for the assessment of pluripotency in these lines. We analysed generated iPSCs for the alkaline phosphatase activity. Since the alkaline phosphatase is a stem cell membrane marker, the elevated expression of this protein is a demonstration of undifferentiated, pluripotent state. The photographs showing cobblestone-like morphology of the obtained iPSCs and results of stainings for the alkaline phosphatase activity are presented in Fig. 2a.

Next, we analysed the established iPSC lines for the presence of pluripotency-associated transcription factors and membrane markers. For this purpose, we conducted the immunofluorescence detection by using anti-NANOG-, anti-OCT3/4-, and anti-C-MYC-specific antibodies to confirm iPSC identity. iPSCs generated from all studied cells were shown to be positive for these factors. Furthermore, we analysed iPSC lines for the pluripotency markers present on their cell membrane. Immunofluorescence analysis presented in Fig. 2a demonstrates the presence of molecular markers characteristic for undifferentiated stem cells and confirms that generated lines are genuine pluripotent cells.

As the ability to differentiate into the cells of the three germ layers is the eminent characteristic of pluripotent cells, we subjected iPSCs generated by episome-based reprogramming to in vitro differentiation studies by allowing them to form EBs. The resulting cells were immunophenotyped for markers characteristic for each particular germ layer. Map2-specific antibody was used to assess the ability of EBs to form ectoderm-derived cells, alpha-smooth muscle actin (αSMA) was used as a mesodermal marker, and Sox17-specific antibody was used to detect differentiated cells of ectoderm origin.

The results of immunostainings were evaluated under the wide-field epifluorescence microscope and presented in Fig. 2b. Altogether, results of the EB-mediated in vitro differentiation experiment show that iPSC derived from somatic cells used in this study demonstrate the potential to generate cells of all three germ layers.

In the last characterization study, we investigated the transcriptional status of the established iPSCs. For this purpose, we compared expression profiles of 94 pluripotency- and lineage-specific genes by using TaqMan-based PCR and by using hPSC Scorecard cloud-based software. In the experiment, we included hESCs and the initial starting material from which corresponding iPSCs were derived as controls.

**Fig. 3:**
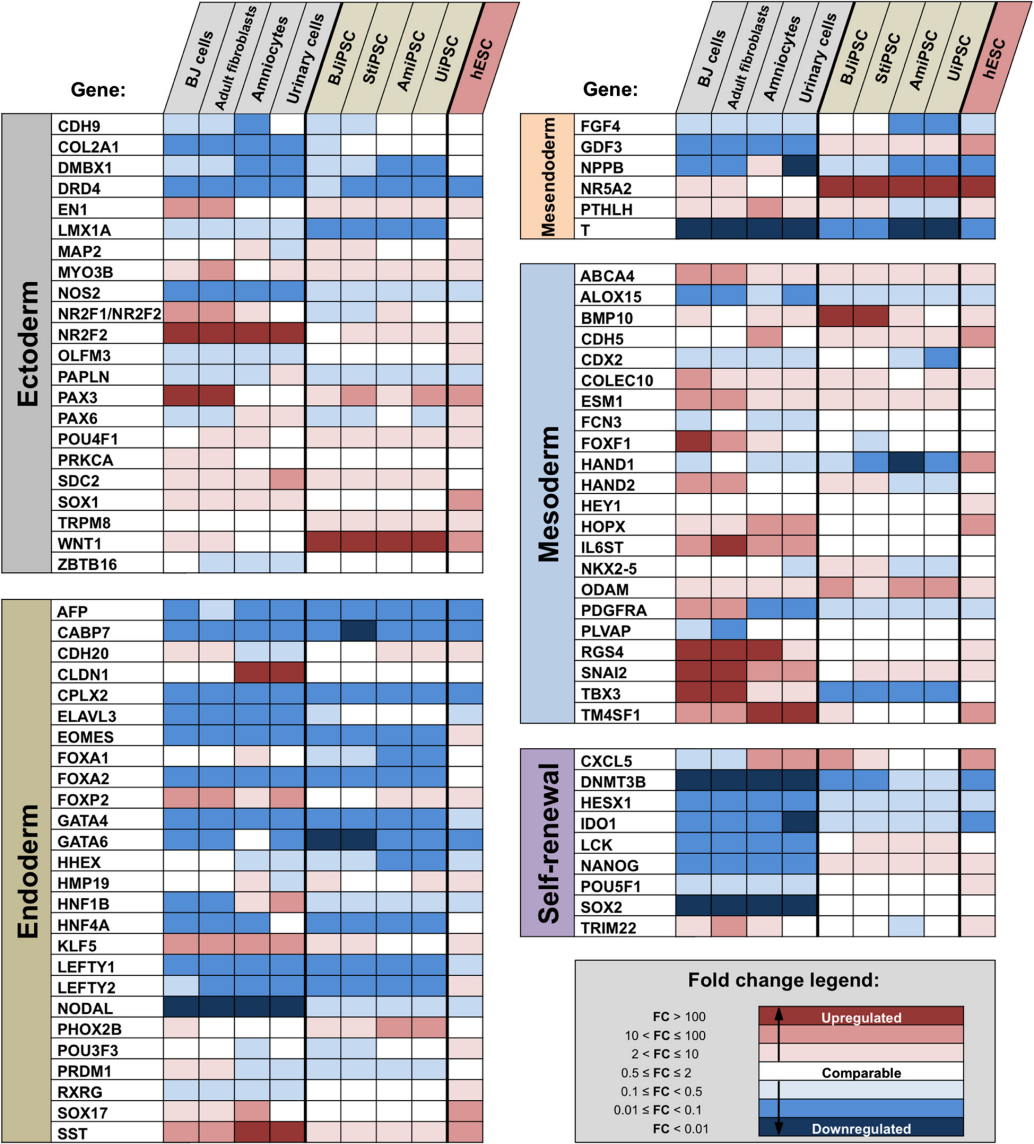
Expression profiles of pluripotency- and lineage-specific genes in established induced pluripotent stem cell lines. Pluripotency and trilineage differentiation potential was assessed with TaqMan Scorecard kit in accordance with the recommendations of the manufacturer. Quantitative polymerase chain reaction readouts were normalised against the pluripotency- and germline-specific controls. Human embryonic stem cell (hESC) line and cells from the initial starting material were used as controls. Data analysis and normalisation were performed by using the cloud-based TaqMan Human Pluripotent Stem Cell Scorecard analysis software. *AmiPSC* amniocyte-derived induced pluripotent stem cell, *BJiPSC* induced pluripotent stem cell derived from BJ cell, *StiPSC* induced pluripotent stem cell derived from scar tissue fibroblast, *UiPSC* induced pluripotent stem cell reprogrammed from urinary epithelial cells

Results of this study, presented as a heat map in Fig. 3, show the expression profiles of pluripotency- and lineage-associated markers, indicating their differentiation propensities. The qPCR experimental data and the pair-wise comparison of the expression levels of factors involved in self-renewal process (Additional file 6: Figure S3B) confirmed the presence of self-renewal factors at levels comparable to undifferentiated ESCs. In addition, the Scorecard algorithm indicated a slight bias of fibroblasts-derived iPSCs toward the mesodermal lineage (Additional file 6: Figure S3B).

Taken together, our data demonstrate that iPSCs derived from fibroblasts, amniocytes, and urinary epithelial cells by means of episomal system were reprogrammed to pluripotency and display functional properties of ESCs.

### Factors influencing induced pluripotent stem cell reprogramming efficiency

Episomal system used for generation of iPSCs in this study has a modular structure and is composed of five plasmids, four of which contain oriP/EBNA-1 elements designed to facilitate long-term expression of introduced transgenes. Since episomes are non-integrating vectors temporarily present in cells undergoing reprogramming, it is possible to deliver several genes in separate oriP/EBNA-1 plasmids for simultaneous expression. It is no longer critical to reduce the number of reprogramming factors due to limited cargo capacity, like in the case of retroviral or certain transposon-based reprogramming systems.

**Fig. 4:**
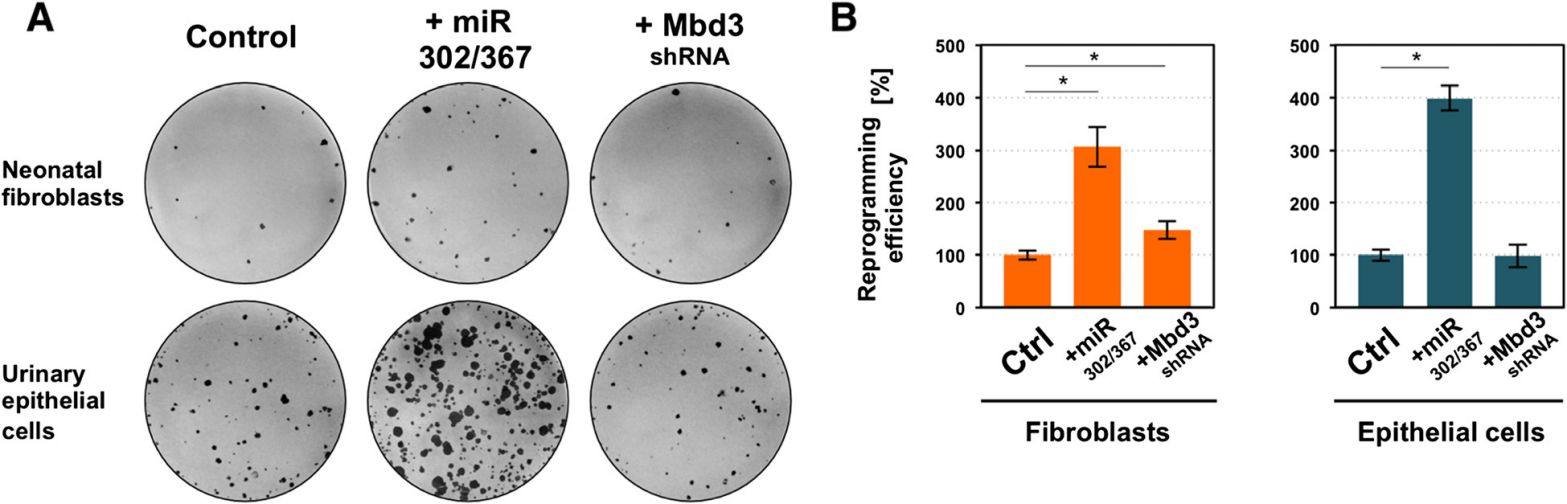
The impact of miR 302/367 overexpression and Mbd3 depletion on induced pluripotent stem cell (iPSC) colony-forming efficiencies. (**a**) Exemplary alkaline phosphatase stainings of iPSCs generated from foreskin neonatal fibroblasts and urinary epithelial cells. Plates labelled as control show cells reprogrammed with episomes carrying OCT3/4, SOX2, KLF4, L-MYC, LIN28, and p53 dominant negative mutant. Cells labelled as + miR 302/367 were additionally transfected with episomal construct comprising mCherry-miR 302/367 cassette driven by cytomegalovirus promoter, whereas + Mbd3 shRNA indicates the cells additionally transfected with episomal vector carrying shRNA against Mbd3 mRNA under control of U6 promoter. (**b**) Summary of the reprogramming experiments with use of vectors carrying miR 302/367 and Mbd3 shRNA constructs. Graphed data show results of triplicate experiments presented as a mean ± standard error of the mean. Asterisks indicate statistically relevant difference between compared samples. *Ctrl* control, *Mbd3* methyl-CpG-binding domain protein 3, *shRNA* short hairpin RNA

Recently, it was demonstrated that upregulation of miR 302/367 and suppression of Mbd3 protein can promote generation of iPSCs. To address the potential of these factors to influence episomal reprogramming, we created two oriP/EBNA-1-based vectors by incorporating mCherry-miR 302/367 cassette for microRNA overexpression and shRNA construct for depletion of endogenous Mbd3 protein. To determine the effect of generated vectors on the expression levels of Mbd3 and miR 302/367 cluster, we performed semi-quantitative end-point RT-PCR on template from BJ neonatal fibroblasts and renal epithelial cells transfected with reprogramming episomes alongside with pCE-DEST (empty vector used as a control), pCE-mCherry-miR302/367, and pCE-shMbd3 episomal plasmids. The result of the experiment, presented in Additional file 7: Figure S4, indeed confirmed the upregulation of miR 302/367 transcript and decrease in Mbd3 expression. An iPSC generation experiment was carried out by using BJ fibroblasts and urinary epithelial cells. These representative cells were chosen on the basis of their high reprogramming efficiencies. Studied cells (8 × 10^4^) were transfected with 2 μg of five plasmids of previously used episomal system and 1 μg of pCE-DEST (empty vector used as a control) or pCE-mCherry-miR302/367 or pCE-Mbd3shRNA. Transfection was performed with FuGENE6 by using a 3:1 reagent-to-DNA ratio. The culture media exchange was done as described in earlier experiments, and after 3 weeks cells were fixed and assessed for alkaline phosphatase activity. The exemplary results of this experiment are presented in Fig. 4a, and the summary of three independent reprogramming assays is shown in Fig. 4b.

The obtained data show that the introduction of miR 302/367 cassettes enhances the reprogramming efficiency threefold for fibroblastic cells and fourfold for urinary epithelial cells. The incorporation of shRNA against Mbd3 protein into the episomal reprogramming system improved the efficiency by 47 % for fibroblasts but shows no effect for epithelial cells.

**Fig. 5:**
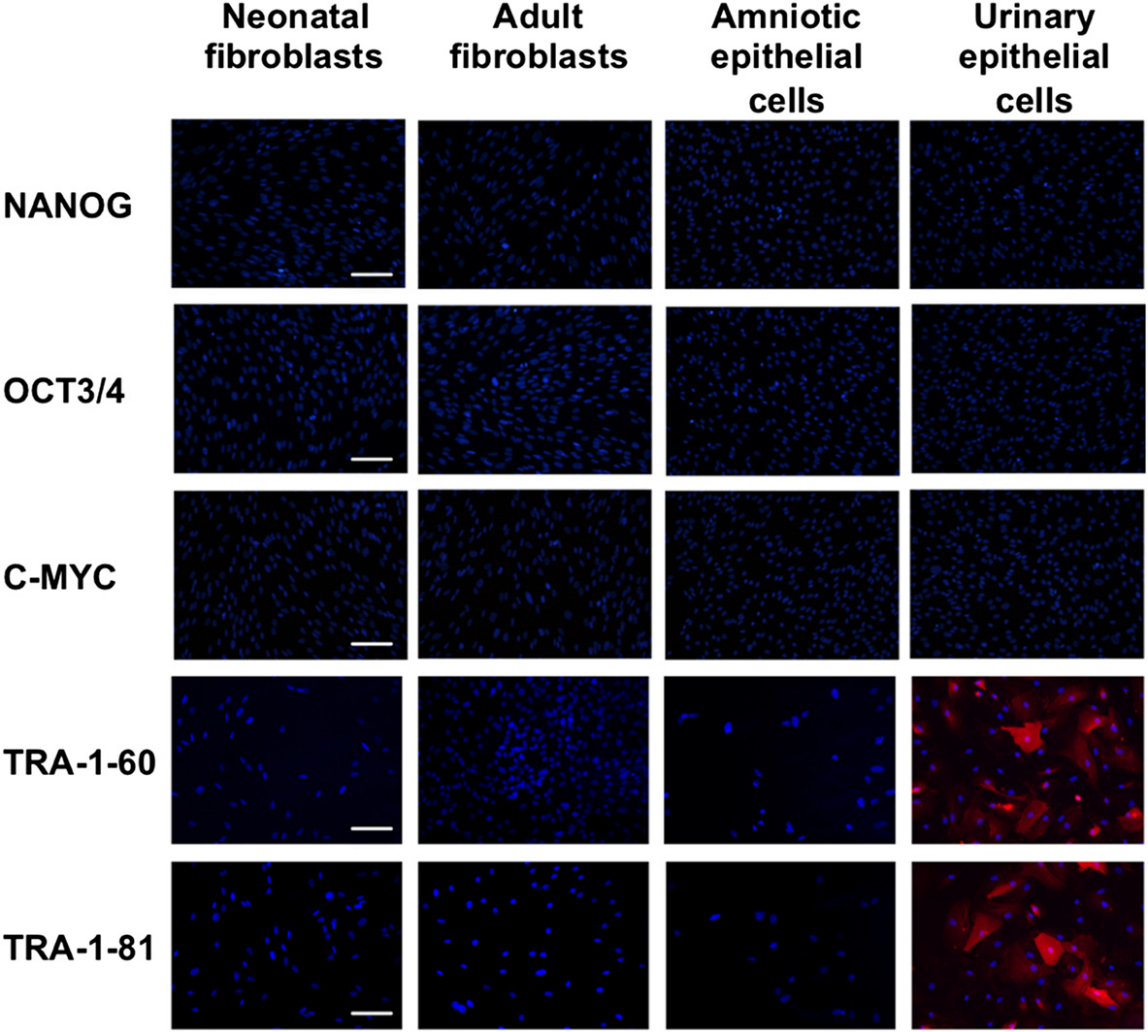
Immunocytochemical detection of pluripotency-associated surface proteins in somatic cells used for generation of induced pluripotent stem cells. Fibroblastic and epithelial cells from the initial starting material were stained with anti-NANOG, anti-OCT3/4, anti-C-MYC, anti-TRA-1-60, and anti-TRA-1-81 antibodies and counterstained with 4ʹ,6-diamidino-2-phenylindole (DAPI). Urinary epithelial cells displayed TRA-1-60 and TRA-1-81 markers on their surface as captured with an Eclipse Ci-S epifluorescence microscope with 20× objective. Scale bar = 100 μm

The additional issue regarding the reprogramming efficiency involves the presence of factors in donor cells required for iPSC generation or facilitating the overcoming of reprogramming barriers or both. The endogenous presence of such factors would obviate the necessity of their overexpression and therefore makes these cells more amenable to reprogramming. We analysed the expression of pluripotency-associated markers used for the generation of iPSCs in this study by means of immunocytochemical stainings. Results of this experiment are presented in Fig. 5.

We could detect the presence of TRA-1-60 and TRA-1-81 membrane proteins in urine-derived cells out of all analysed pluripotency-related genes. The presence of these markers correlates with the highest iPSC generation efficiency obtained for urinary epithelial cells, as shown in Fig. 1d.

## Discussion

With the recent development of nuclear reprogramming technology, the idea of patient-personalized therapies and replacement of diseased or damaged organ tissues came to the horizon of clinical reality. In this work, we address the question of the applicability of safer, non-viral episomal reprogramming to derive fully functional iPSCs from human cells of various origin. The aim of the current study was to establish whether donor cells obtained from different starting material could offer certain advantages in terms of reprogramming yield and quality of generated iPSCs. Cells used in this work were derived from neonatal foreskin and adult scar tissue as well as from urine and amniotic fluid. Urinary epithelial cells and amniocytes were described previously as a donor material useful for efficient generation of iPSCs [28, 31]; however, the reprogramming potential of these two cell types has never been compared in the same experimental setup.

Direct reprogramming of somatic cells into the pluripotent state can be accomplished by forced expression of defined transcription factors. Cells can be genetically modified by using viral, physical, or chemical approaches. The chemical methods of transfection are broadly used and are considered to be safe and cost-effective, as they require neither special equipment nor a laboratory facility dedicated for work with biohazard agents. As the efficient gene transfer is a prerequisite condition for successful generation of iPSCs, we decided to find optimal conditions for transfer of episomes carrying reprogramming factors.

In the first part of our work, we carried out reporter gene experiments and cytotoxicity measurements to find out which compounds and transfection conditions lead to the highest expression of introduced transgenes and result in lowest cytotoxic effect in analysed samples. Using four chemical compounds and commercially available transfection reagents, we established that FuGENE6 reagent complexed with episomal DNA at a 3:1 ratio was the most suitable for further studies.

In subsequent experiments with application of oriP/EBNA-1 episomal system, we conducted a comparative analysis of reprogramming efficiencies of adult and neonatal fibroblasts and of cells of epithelial origin derived from urine and amniotic fluid. The reprogramming assays carried out in feeder-free conditions demonstrated the feasibility of episome-mediated iPSC generation. By applying the same reprogramming procedure, we were able to compare iPSC forming efficiencies across the donor samples of different origin and cellular morphology. The reprogramming process was far more efficient in the case of cells of epithelial origin (10–100-fold) than in fibroblastic cells. Urine-derived epithelial cells were reprogrammed to iPSCs with approximately 1.5 % efficiency, whereas cells isolated from amniotic fluid displayed efficiencies approaching 0.1 %. The type of somatic cells used for deriving iPSCs may influence the efficiency of the reprogramming. Molecular transition of many cells undergoing the reprogramming process involves the changes in morphology and alteration of the expression of certain transcription factors known as mesenchymal-to-epithelial transition (MET). Previously, it was shown that retroviral transduction of keratinocytes with OSKM canonical factors results in 1 % of reprogramming efficiency, which was two orders of magnitude higher than in the case of fibroblasts (0.01 %) [32]. In addition, in reprogrammed keratinocytes, iPSCs emerged 10 days after infection, in contrast to 3–4 weeks for fibroblasts. Keratinocytes display similar properties to pluripotent cells in terms of the tight, cell-to-cell morphology and high level of cadherin expression. It has been proposed that since both pluripotent cells and keratinocytes are epithelial cells and probably have a similar epigenetic state, keratinocytes do not have to undergo the MET required for fibroblasts [32]. Given that epithelial cells display accelerated reprogramming kinetics and that morphologically distinct iPSCs appear approximately 1–2 weeks earlier than fibroblasts being reprogrammed, our results provide the independent confirmation that alleviation of the MET requirement in the iPSC generation process is a more general feature of cells of epithelial origin.

Regarding the two lines of fibroblastic origin, we observed higher iPSC colony-forming efficiencies for neonatal fibroblasts (0.011 %) than for adult scar tissue cells (0.003 %). It is important to mention that using transiently introduced reprogramming episomes in BJ cell line we could obtain the same reprogramming efficiency, as earlier reported for retroviral vectors [2]. Fibroblasts involved in scar tissue formation are specialized cells displaying fibroblastic and smooth muscle cell properties (miofibroblasts). It is not clear why scar tissue fibroblasts exhibit lower iPSC formation efficiency than neonatal fibroblast cells. This difference in term of reprogramming efficiency cannot be attributed simply to the initial passage number, since the both cell lines from which the iPSCs were derived could be maintained in culture for couple of weeks without any morphological signs of cellular senescence. In addition, it appears that the gene delivery was not the limiting factor in the reprogramming process, as scar tissue fibroblasts, among all cells used in this study, actually provided the highest transfection efficiencies in reporter gene assays, so the additional molecular mechanisms must be involved.

However, the issue of cellular senescence is valid for epithelial cells. We found that it was essential to use cells at the earliest passage possible because, in the case of donor cell population displaying morphological signs of senescence, only a few iPSC colonies could be derived. We observed that for amniocytes and urinary cells, iPSC formation substantially declines after approximately the fifth passage from the derivation of initial colonies of these cells.

Yu et al. [19] reported reprogramming with episomes, but the “gene cocktail” used in their experiments included NANOG and SV40 large T antigen, whereas in the episomes used in our studies, NANOG was omitted and the dominant negative mutant of p53, instead of SV40 large T antigen, was used to overcome apoptosis and senescence. In this report, human dermal fibroblasts could be reprogrammed with efficiencies approaching 0.006 %.

The setup of the reprogramming experiments in our study was comparable to those developed and evaluated by Okita et al. [20, 21], as we used a “pCE” set of reprogramming episomes described in these studies, but some modifications in the procedure were applied. Firstly, we plated cells on the basement membrane protein matrix and not the feeder cell layer. In addition, episomal vectors carrying reprogramming genes were delivered to the cells being reprogrammed by means of chemical transfection in contrast to electroporation, which was used in the mentioned studies. iPSC induction efficiencies described by Okita et al. for neonatal foreskin fibroblasts were comparable to our data (0.011 % for BJ cells and 0.015 % described in [20]); however, in our studies, ESC-like colonies started to form 7 days later.

In this study, we demonstrate that iPSC lines established from BJ cells, scar tissue fibroblasts, amniocytes, and renal epithelial cells by using episomal plasmids displayed typical ESC-like morphology. In addition, immunofluorescence and colorimetric assays indicated that all iPSC lines expressed pluripotency antigens, including alkaline phosphatase, TRA-1-60, TRA-1-81, and OCT3/4 transcription factor. For other nuclear factors, we could confirm the endogenous expression of C-MYC and NANOG genes. It is important to point out that the coding sequences of the latter two genes were not included in the episomal system as components of reprogramming factors.

Results of the qPCR experiments also confirmed the presence of self-renewal factors in iPSC lines established from fibroblastic and epithelial somatic cells. The expression levels of pluripotency genes were comparable to undifferentiated ESCs. Furthermore, the Scorecard algorithm indicated the bias of fibroblast-derived iPSCs toward the mesodermal lineage. Such a propensity could be a result of particular chromatin structure and remnants of epigenetic memory of donor cells, which for some reason was able to withstand the nuclear reprogramming that brought these cells to pluripotency. Interestingly, such a tendency was not observed for iPSCs generated from renal epithelial cells, which also originated from mesodermal tissue. It suggests that, in addition to demonstrating higher reprogramming efficiency and enhanced temporal kinetics, iPSCs derived from epithelial cells demonstrate better quality in terms of their similarity to the epigenetic state of pluripotent cells. Nevertheless, it is important to mention that in EB-mediated differentiation assays we could easily establish cells of all three germline layers from iPSCs generated from neonatal and adult fibroblasts, amniocytes, and urinary epithelial cells. It was demonstrated by the presence of MAP2-, αSMA-, and SOX17-positive cells obtained in the course of in vitro differentiation experiments.

In this work, we also aimed to investigate the influence of certain factors on the episomal reprogramming. Recently, it was demonstrated that certain microRNA clusters, especially these including miR 302/367, dramatically promote generation of iPSCs when co-expressed with canonical OSKM transcription factors and even completely substitute OCT3/4, SOX2, KLF4, and C-MYC, which earlier were considered indispensable for efficient reprogramming [33]. In addition, Jacob Hanna’s group has reported the achievement of 96 % of iPSC colony formation efficiency in murine somatic cells by knockout of methyl-binding protein 3 (Mbd3), a component of nucleosome remodelling complex NuRD. As the above findings offer new approaches for increasing reprogramming efficiencies in the generation of iPSCs, we addressed the question of whether the incorporation of miR 302/367 cassette or Mbd3 shRNA sequence into the episomal system could also have an effect on its efficiency and reprogramming speed.

To explore the potential of additional vectors to influence the reprogramming process, we carried out additional iPSC generation experiments by using BJ fibroblasts and urinary epithelial cells. For the miR 302/367 cassette, we observed that the overexpression of this cluster enhances iPSC colony-forming efficiencies three times in reprogrammed fibroblasts and four times during derivation of pluripotent cells from urinary epithelial cells.

MicroRNAs are engaged in regulation of gene expression in certain developmental and physiological processes. Recent studies indicate that microRNAs influence cell fate and play an essential role in control of self-renewal and differentiation of stem cells (reviewed in [34, 35]). Certain regulatory clusters, especially miR 290/295 and miR 302/367, were shown to significantly facilitate the generation of iPSCs from murine and human cells. MicroRNAs may promote nuclear reprogramming to some extent by decreasing the expression of transforming growth factor-beta (TGF-β) receptor (demonstrated for miR 302), as this signalling pathway promotes epithelial-to-mesenchymal transition [36], or providing anti-apoptotic and anti-senescence effect associated with miR 290/295 [37]. Furthermore, there are consensus recognition motifs of NANOG, OCT3/4, and SOX2 transcription factors found within miR 302/367 promoter [38]. On the other hand, miR 302/367 also appears to inhibit certain repressors of pluripotency factors, thereby providing reciprocal regulation, as the TALEN-mediated excision of miR 302/367 cluster has been shown to render human fibroblasts incapable of iPSC generation [39].

Our results demonstrated that the incorporation of shRNA against Mbd3 protein into the episomal system improved the efficiency by 47 % for fibroblasts but that it showed no effect for epithelial cells. This result led us to the conclusion that repression of the NuRD remodelling complex facilitates MET in cells undergoing reprogramming. It has been reported [40] that nearly 100 % iPSC colony formation efficiency was achieved in cells with stably depleted Mbd3 protein. However, these efficiencies could be obtained only in cells established from the Mbd3 knockout animal model, whereas transient downregulation in wild-type fibroblasts acquired by transfection of small interfering RNA (siRNA) resulted in approximately 18 % enhancement of the reprogramming efficiency. The improved iPSC forming efficiency in our study can most likely be attributed to the retention of episomes carrying shRNA cassette after cell divisions, resulting in prolonged presence of the interfering constructs.

In this work, we found that the inclusion of vectors carrying miR 302/367 or shRNA against Mbd3 was not an absolute necessity to obtain a reasonable number of iPSC colonies from most of the reprogrammed cells. Nevertheless, we think that the incorporation of these additional vectors may provide an advantage in certain circumstances, such as dealing with a small number of cells from the donor material or in conditions where cells to be reprogrammed show poor quality in terms of high passage number.

The additional issue regarding the reprogramming efficiency involves the presence of factors in donor cells, which are required for iPSC generation or facilitating the overcoming of reprogramming barriers or both. The endogenous presence of such factors would obviate the necessity of their expression *in trans* and therefore would make these cells more amenable to nuclear reprogramming. The most prominent example of such cellular status includes neural stem cells. As they continuously expresses SOX2, KLF4, and C-MYC, they can be converted to iPSCs by the ectopic expression of a single OCT3/4 factor [24]. In our work, we reported the endogenous expression of TRA-1-60 and TRA-1-81 epitopes of podocalyxin on membranes of renal epithelial cells. The presence of this protein is typically a hallmark of undifferentiated pluripotent cells. In addition, the expression of podocalyxin has been confirmed in other stem cells, including hematopoietic multipotent progenitors [41, 42], and these cells were previously used for relatively efficient generation of iPSCs [43]. Podocalyxin, also described as TRA-1-60 and TRA-1-81 in the literature, is a negatively charged membrane-bound sialoglycoprotein. One could speculate that podocalyxin interactions with positively charged RGD (Arg-Gly-Asp) moieties of extracellular proteins required for the maintenance of undifferentiated pluripotent cells [44–46]; especially, laminin, fibronectin, and vitronectin may promote the acquisition of pluripotent identity.

Although the role of TRA-160 and TRA-1-81 markers in the process of achieving pluripotency is poorly understood and needs to be further studied, we could demonstrate that the expression of podocalyxin in the population of donor cells used for reprogramming experiments clearly correlates with enhanced reprogramming efficiency.

## Conclusions

In this study, we carried out a comparative analysis of iPSC generation efficiencies in fibroblasts and epithelial cells transiently transfected with episomal plasmids. We determined that colony-forming efficiencies are highest for donor samples derived from epithelial cells. Observed discrepancies may be due to the fact that epithelial cells omit MET and by that means enhance efficiencies and accelerate the process of reprogramming. We demonstrated that reprogramming efficiency of the oriP/EBNA-1 system could be further improved. In particular, the upregulation of miR 302/367 cluster expression enhances reprogramming of fibroblastic and epithelial cells, whereas Mbd3 downregulation improved the reprogramming of fibroblasts only. Furthermore, we confirmed the presence of TRA-1-60 and TRA-1-81 surface proteins on the membranes of urinary epithelial cells. The possible role of these proteins in the process of reprogramming needs to be elucidated in detail since their expression in the population of donor cells correlates with highly elevated iPSC forming efficiency.

## Additional files

Additional file 1: Table S1. Primers used for DNA amplification and adapter assembly. Additional file 2: Table S2. Antibodies used for immunocytochemical analysis. Additional file 3: Figure S1. Comparison of transfection efficiencies of transfection reagents:DNA complexes at different ratios in BJ cells. The episomal expression plasmid containing *EmGFP* gene was transiently introduced into human neonatal fibroblast cell line. Transfection with reagent:DNA complexes was carried out in the presence of serum by using various amounts of transfection reagents. Two days after transfection, cells were fixed with paraformaldehyde, counterstained with 4ʹ,6-diamidino-2-phenylindole (DAPI), and analysed with an epifluorescence microscope. Scale bar = 100 μm. *PEI* polyethylenimine. Additional file 4: Figure S2. Comparative analysis of the reporter gene expression and transfection-associated toxicity in neonatal, adult fibroblasts, amniotic fluid, and urinary epithelial cells. (**a**) Secreted alkaline phosphatase activity was measured for the constant number of plated cells transiently transfected with episomal plasmid. CAGGS promoter driven reporter construct was introduced into the studied cells by using varying amounts of FuGENE6 transfection reagent. Graphed data are presented as mean ± standard error of the mean (*n* = 3). (**b**) Dynamic monitoring of the cell number and adhesion using xCELLigence system. Six thousand of each of the studied cells were seeded on one well of the assay plate and transfected with plasmid by using FuGENE6 reagent. Four different ratios were tested to examine the toxic effect elicited by the compounds used for episomal delivery. The cytotoxic effect was monitored for 72 h, and cell indexes are presented as a mean of three independent counts. Additional file 5: Table S3. Summary of induced pluripotent stem cell generation experiments from cells of different origin. Additional file 6: Figure S3. Comparison of the expression levels of pluripotency-associated genes and lineage-specific differentiation propensities in established induced pluripotent stem cell (iPSC) lines. (a) Scatter plots comparing threshold cycle (C_T_) values of genes involved in self-renewal process. The pair-wise comparison includes generated iPSCs, original cells from which pluripotent cells were induced, and human embryonic stem cells (hESC) used as a reference. Pearson correlation coefficient (R^2^) describes the similarity of the corresponding cells to hESCs in terms of the pluripotency-related gene expression. (b) Scorecard algorithm prediction displaying lineage identity (for the initial starting cells) and self-renewal properties and the trilineage differentiation propensities of the generated iPSC lines and hESCs. *AmiPSC* amniocyte-derived induced pluripotent stem cell, *BJiPSC* induced pluripotent stem cell derived from BJ cell, *StiPSC* induced pluripotent stem cell derived from scar tissue fibroblast, *UiPSC* induced pluripotent stem cell reprogrammed from urinary epithelial cells. Additional file 7: Figure S4. Confirmation of upregulation of the miR 302/367 and downregulation of Mbd3 expression by episomal vectors. BJ cells and urinary epithelial cells were transfected with pCE-DEST (Ctrl), pCE-mCherry-miR 302/367, and pCE-shMbd3 episomes. One week after transfection, total RNAs were isolated and reverse-transcribed. The outcome of transfection was confirmed by semi-quantitative end-point reverse transcription-polymerase chain reaction by using Mdb3 Left, Mbd3 Right, miR 302/367 Left, and miR 302/367 Right primers. 18S rRNA was used for DNA input normalisation. *Mbd3* Methyl-CpG-binding domain protein 3.

## Abbreviations

αSMA: Alpha-smooth muscle actin
BCIP: 5-bromo-4-chloro-3-indolyl phosphate
CI: Cell index
DMEM: Dulbecco’s modified Eagle’s medium
DS: Dyad symmetry
EB: Embryoid body
EBNA-1: Epstein-Barr virus nuclear antigen-1
EBV: Epstein-Barr virus
EDTA: Ethylenediaminetetraacetic acid
ESC: Embryonic stem cell
FCS: Foetal calf serum
hESC: Human embryonic stem cell
hPSC: Human pluripotent stem cell
iPSC: Induced pluripotent stem cell
Mbd3: Methyl-CpG-binding domain protein 3
MET: Mesenchymal-to-epithelial transition
mSEAP: Murine secreted alkaline phosphatase
NBT: Nitro-blue tetrazolium
NEAA: Non-essential amino acid
oriP: Origin of replication
OSKM: Oct3/4, Sox2, Klf4, Myc
PBS: Phosphate-buffered saline
PCR: Polymerase chain reaction
PEI: Polyethylenimine
PFA: Paraformaldehyde
qPCR: Quantitative polymerase chain reaction
REGM: Renal Cell Growth Medium
RTCA: Real-Time Cell Analyzer
SEAP: Secreted alkaline phosphatase
SEM: Standard error of the mean
shRNA: Short hairpin RNA
SV40: Simian virus 40
TBS-T: Tris-buffered 0.9 % saline supplemented with 0.05 % of Tween-20
