# Supplementary figures and images for: Generation of human iPSCs from cells of fibroblastic and epithelial origin by means of the oriP/EBNA-1 episomal reprogramming system
Source: Stem Cell Res Ther. 2015 Jun 19;6(1):122. doi: 10.1186/s13287-015-0112-3 (PMC4515927; doi:10.1186/s13287-015-0112-3)

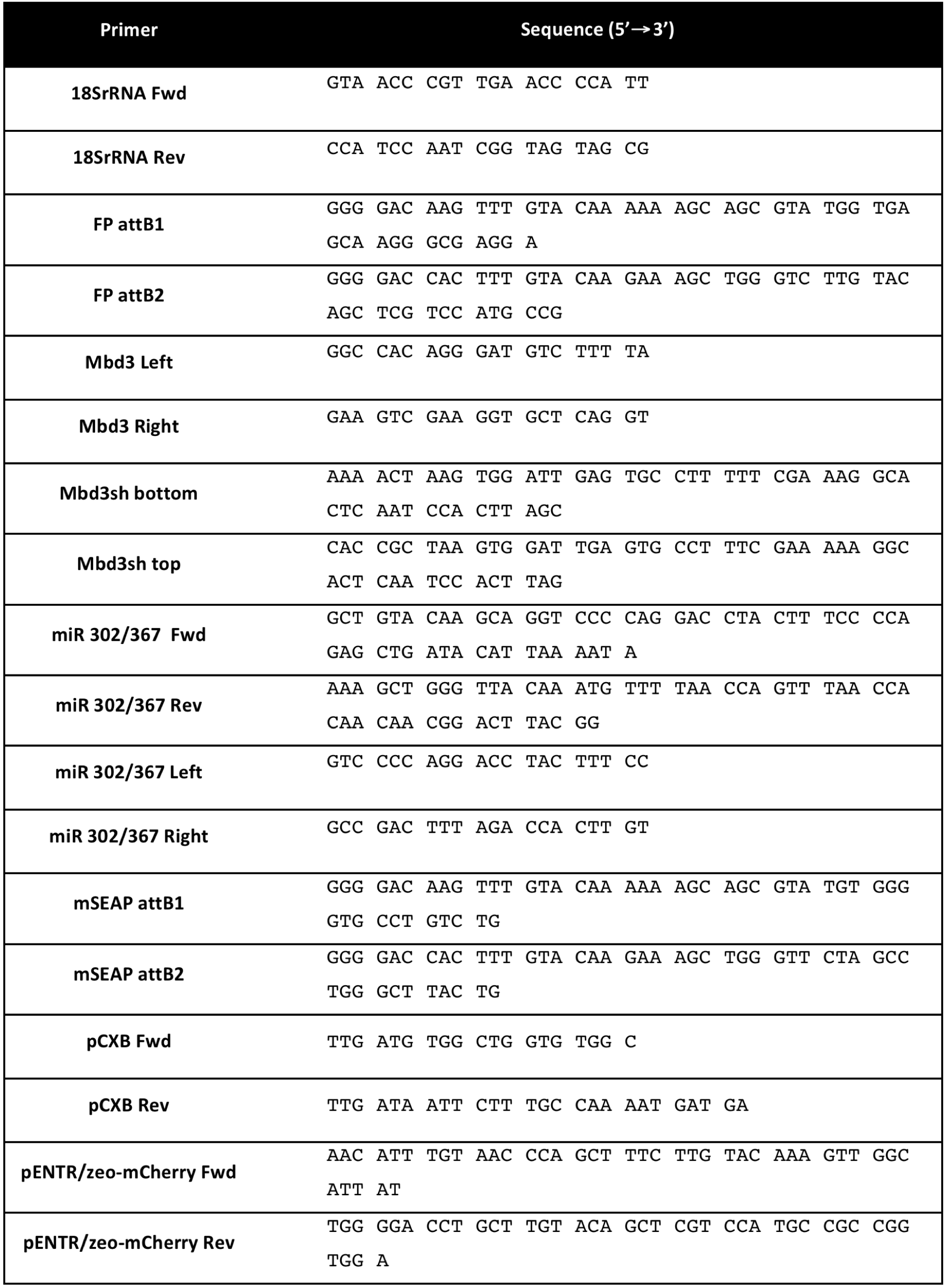

Supplement: Additional file 1: Table S1. — Primers used for DNA amplification and adapter assembly. [file 13287_2015_112_MOESM1_ESM.tiff]

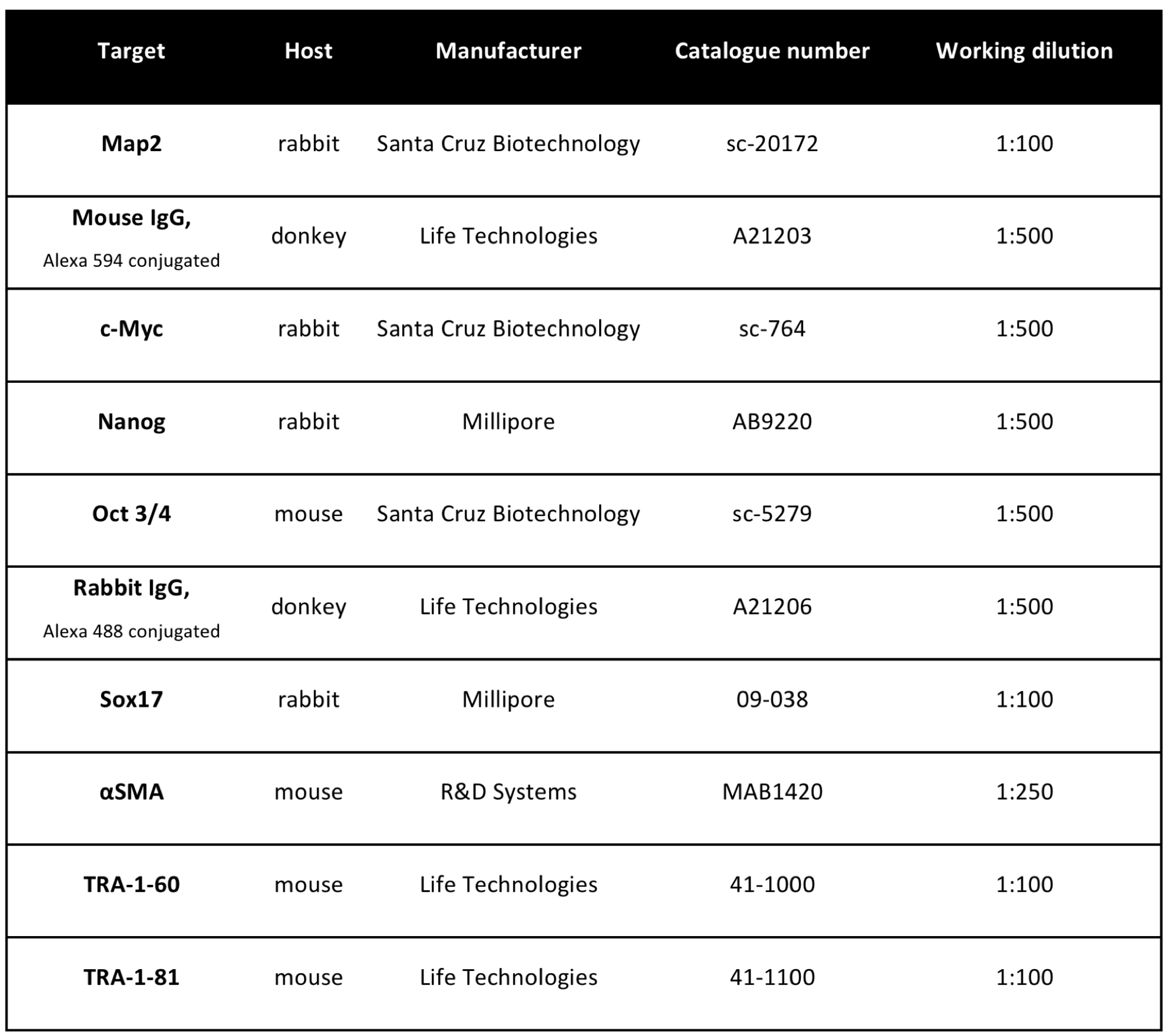

Supplement: Additional file 2: Table S2. — Antibodies used for immunocytochemical analysis. [file 13287_2015_112_MOESM2_ESM.tiff]

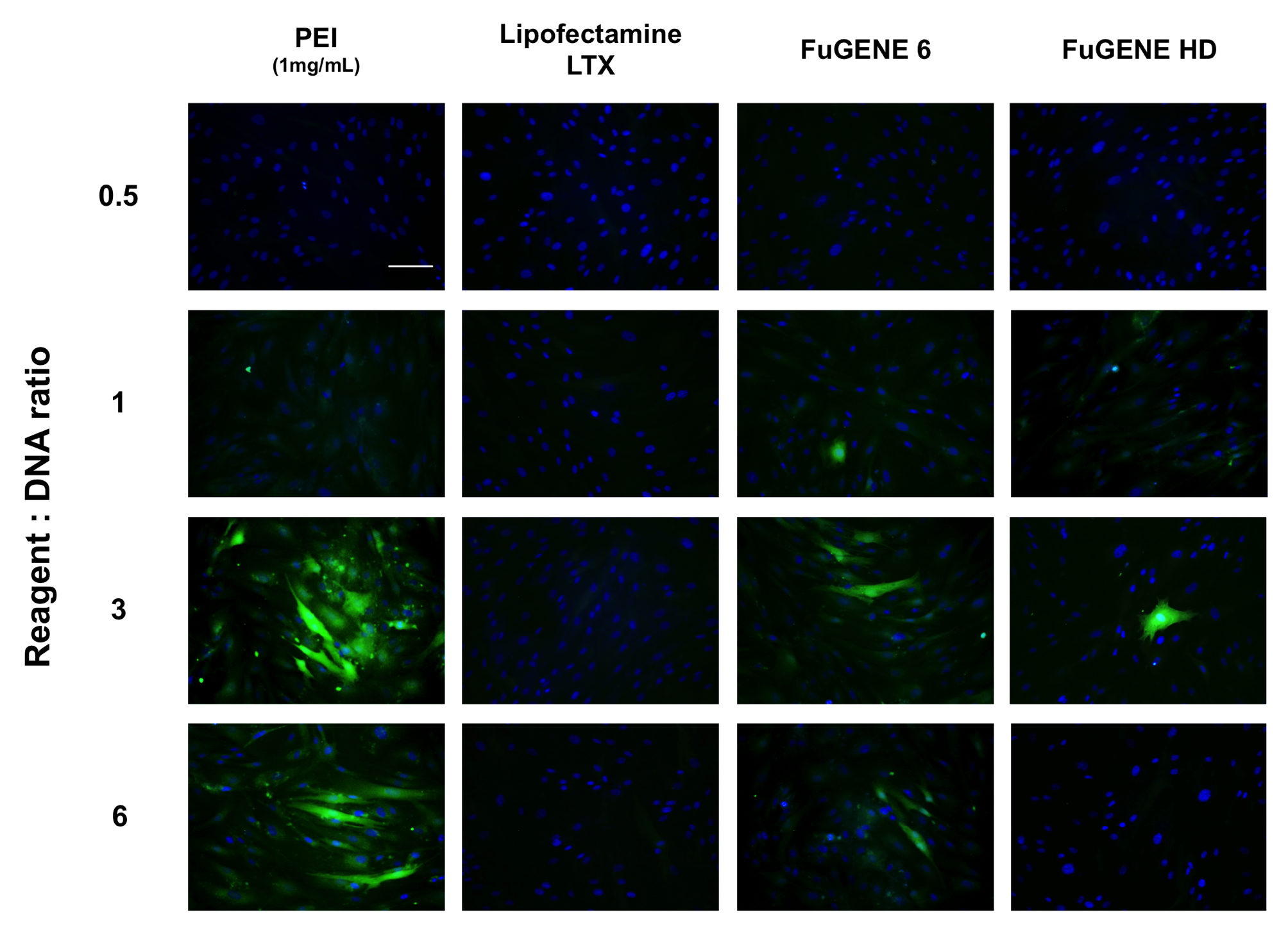

Supplement: Additional file 3: Figure S1. — Comparison of transfection efficiencies of transfection reagents:DNA complexes at different ratios in BJ cells. The episomal expression plasmid containing EmGFP gene was transiently introduced into human neonatal fibroblast cell line. Transfection with reagent:DNA complexes was carried out in the presence of serum by using various amounts of transfection reagents. Two days after transfection, cells were fixed with paraformaldehyde, counterstained with 4ʹ,6-diamidino-2-phenylindole (DAPI), and analysed with an epifluorescence microscope. Scale bar = 100 μm. PEI polyethylenimine. [file 13287_2015_112_MOESM3_ESM.tiff]

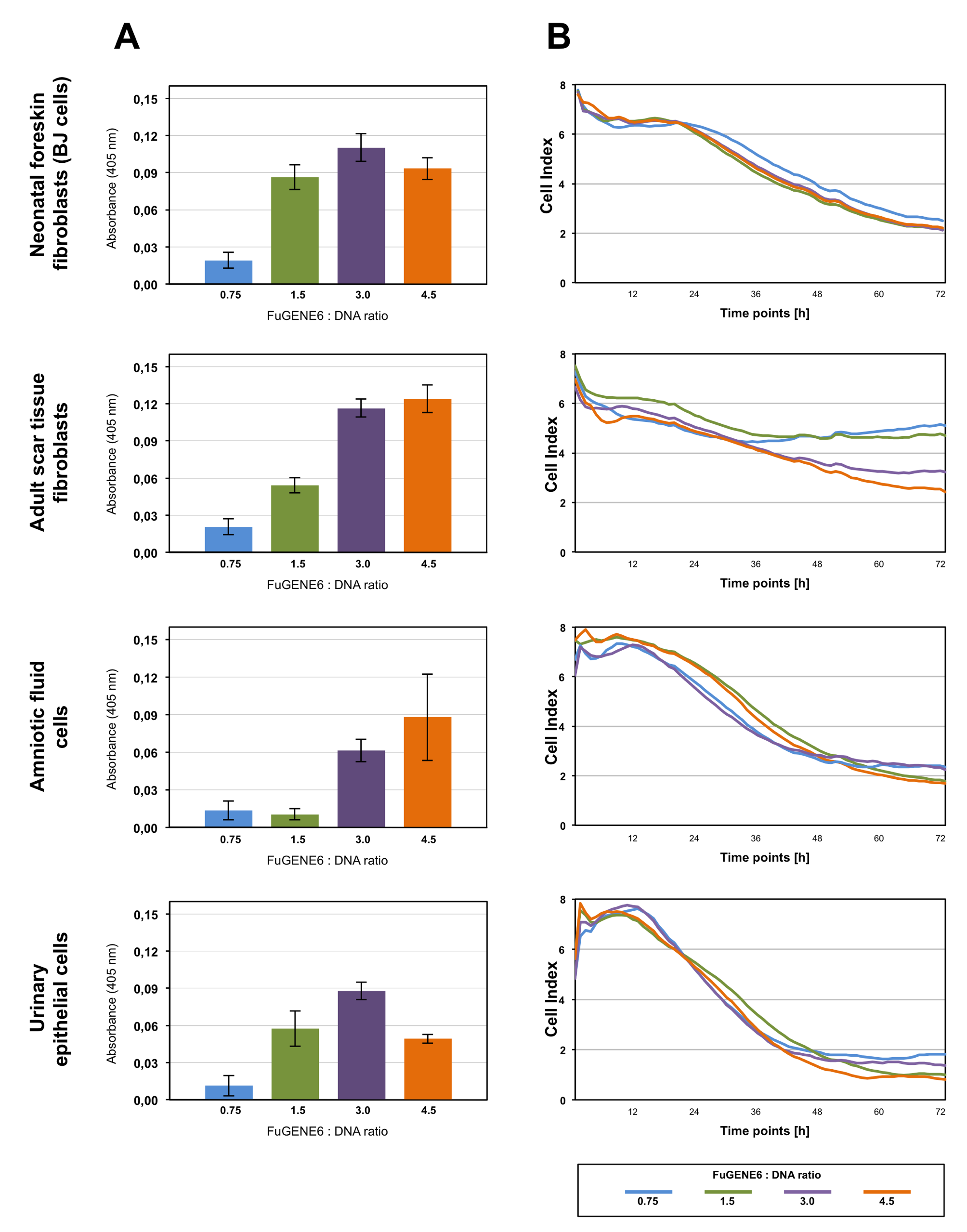

Supplement: Additional file 4: Figure S2. — Comparative analysis of the reporter gene expression and transfection-associated toxicity in neonatal, adult fibroblasts, amniotic fluid, and urinary epithelial cells. (a) Secreted alkaline phosphatase activity was measured for the constant number of plated cells transiently transfected with episomal plasmid. CAGGS promoter driven reporter construct was introduced into the studied cells by using varying amounts of FuGENE6 transfection reagent. Graphed data are presented as mean ± standard error of the mean (n = 3). (b) Dynamic monitoring of the cell number and adhesion using xCELLigence system. Six thousand of each of the studied cells were seeded on one well of the assay plate and transfected with plasmid by using FuGENE6 reagent. Four different ratios were tested to examine the toxic effect elicited by the compounds used for episomal delivery. The cytotoxic effect was monitored for 72 h, and cell indexes are presented as a mean of three independent counts. [file 13287_2015_112_MOESM4_ESM.tiff]

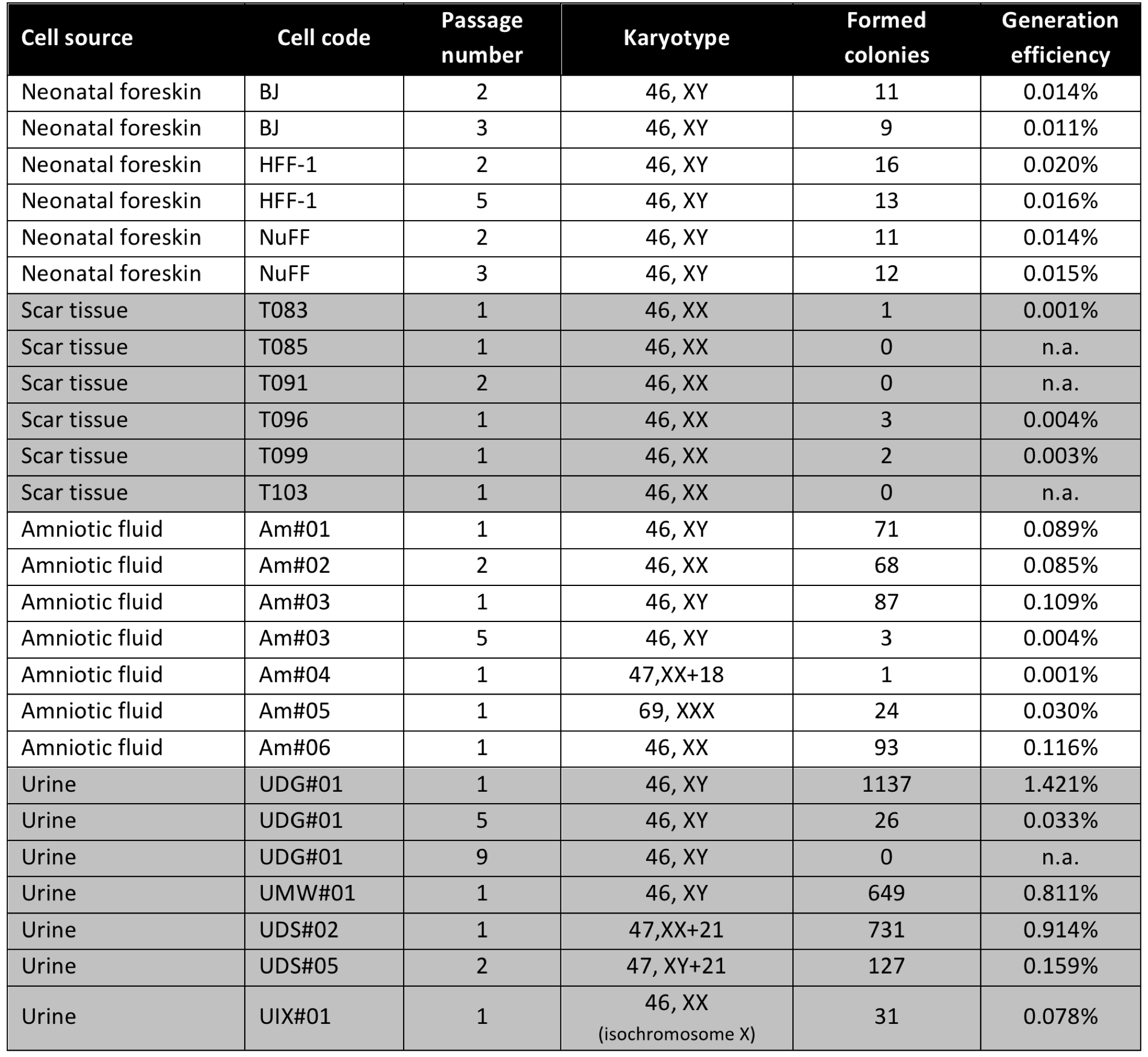

Supplement: Additional file 5: Table S3. — Summary of induced pluripotent stem cell generation experiments from cells of different origin. [file 13287_2015_112_MOESM5_ESM.tiff]

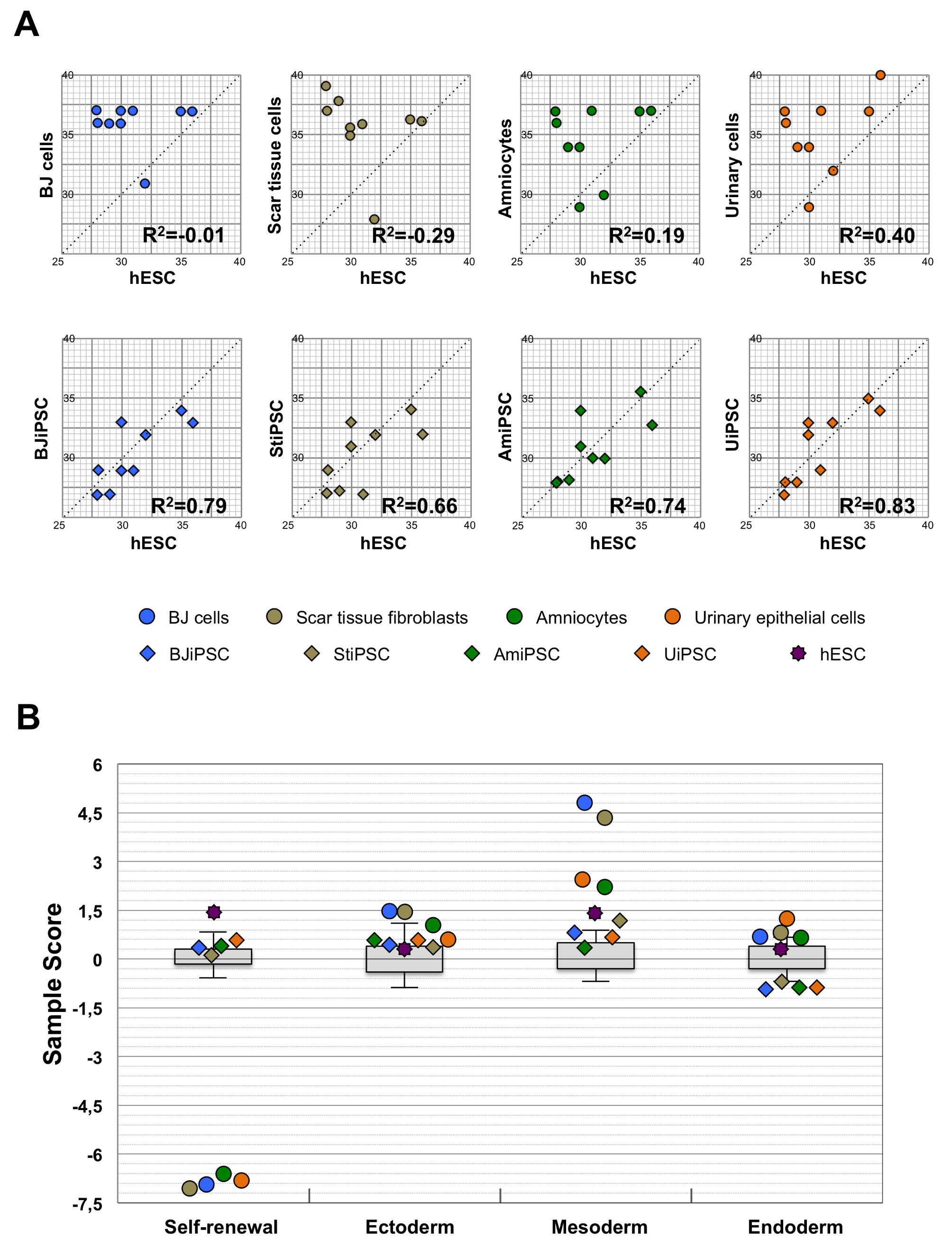

Supplement: Additional file 6: Figure S3. — Comparison of the expression levels of pluripotency-associated genes and lineage-specific differentiation propensities in established induced pluripotent stem cell (iPSC) lines. (a) Scatter plots comparing threshold cycle (CT) values of genes involved in self-renewal process. The pair-wise comparison includes generated iPSCs, original cells from which pluripotent cells were induced, and human embryonic stem cells (hESC) used as a reference. Pearson correlation coefficient (R2) describes the similarity of the corresponding cells to hESCs in terms of the pluripotency-related gene expression. (b) Scorecard algorithm prediction displaying lineage identity (for the initial starting cells) and self-renewal properties and the trilineage differentiation propensities of the generated iPSC lines and hESCs. AmiPSC amniocyte-derived induced pluripotent stem cell, BJiPSC induced pluripotent stem cell derived from BJ cell, StiPSC induced pluripotent stem cell derived from scar tissue fibroblast, UiPSC induced pluripotent stem cell reprogrammed from urinary epithelial cells. [file 13287_2015_112_MOESM6_ESM.tiff]

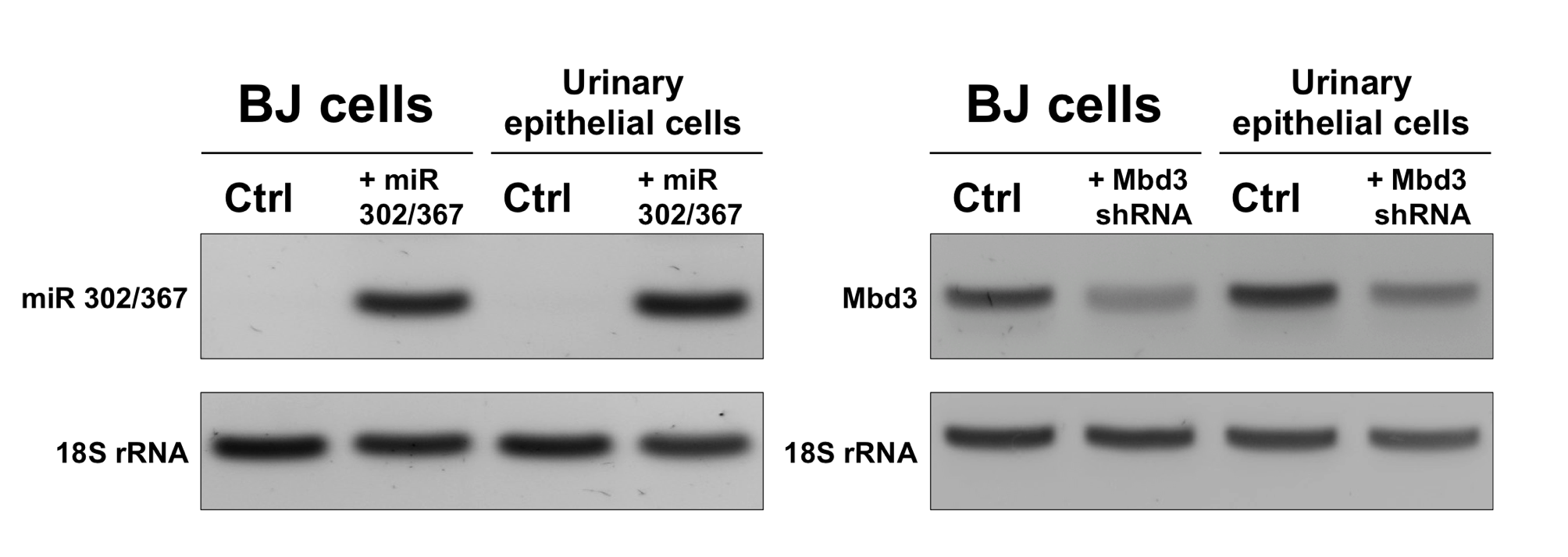

Supplement: Additional file 7: Figure S4. — Confirmation of upregulation of the miR 302/367 and downregulation of Mbd3 expression by episomal vectors. BJ cells and urinary epithelial cells were transfected with pCE-DEST (Ctrl), pCE-mCherry-miR 302/367, and pCE-shMbd3 episomes. One week after transfection, total RNAs were isolated and reverse-transcribed. The outcome of transfection was confirmed by semi-quantitative end-point reverse transcription-polymerase chain reaction by using Mdb3 Left, Mbd3 Right, miR 302/367 Left, and miR 302/367 Right primers. 18S rRNA was used for DNA input normalisation. Mbd3 Methyl-CpG-binding domain protein 3. [file 13287_2015_112_MOESM7_ESM.tiff]
